# Spatial Bistability Generates *hunchback* Expression Sharpness in the *Drosophila* Embryo

**DOI:** 10.1371/journal.pcbi.1000184

**Published:** 2008-09-26

**Authors:** Francisco J. P. Lopes, Fernando M. C. Vieira, David M. Holloway, Paulo M. Bisch, Alexander V. Spirov

**Affiliations:** 1 Department of Applied Mathematics, Stony Brook University, Stony Brook, New York, United States of America; 2 Center for Developmental Genetics, Stony Brook University, Stony Brook, New York, United States of America; 3 Instituto de Biofisica, Universidade Federal do Rio de Janeiro, Rio de Janeiro, Brazil; 4 Instituto de Quimica, Universidade de Brasilia, Brasilia, Brazil; 5 Mathematics Department, British Columbia Institute of Technology, Burnaby, British Columbia, Canada; 6 Chemistry Department, University of British Columbia, Vancouver, British Columbia, Canada; 7 Biology Department, University of Victoria, Victoria, British Columbia, Canada; Duke University, United States of America

## Abstract

During embryonic development, the positional information provided by concentration gradients of maternal factors directs pattern formation by providing spatially dependent cues for gene expression. In the fruit fly, *Drosophila melanogaster*, a classic example of this is the sharp on–off activation of the *hunchback* (*hb*) gene at midembryo, in response to local concentrations of the smooth anterior–posterior Bicoid (Bcd) gradient. The regulatory region for *hb* contains multiple binding sites for the Bcd protein as well as multiple binding sites for the Hb protein. Some previous studies have suggested that Bcd is sufficient for properly sharpened Hb expression, yet other evidence suggests a need for additional regulation. We experimentally quantified the dynamics of *hb* gene expression in flies that were wild-type, were mutant for *hb* self-regulation or Bcd binding, or contained an artificial promoter construct consisting of six Bcd and two Hb sites. In addition to these experiments, we developed a reaction–diffusion model of *hb* transcription, with Bcd cooperative binding and *hb* self-regulation, and used Zero Eigenvalue Analysis to look for multiple stationary states in the reaction network. Our model reproduces the *hb* developmental dynamics and correctly predicts the mutant patterns. Analysis of our model indicates that the Hb sharpness can be produced by spatial bistability, in which *hb* self-regulation produces two stable levels of expression. In the absence of self-regulation, the bistable behavior vanishes and Hb sharpness is disrupted. Bcd cooperative binding affects the position where bistability occurs but is not itself sufficient for a sharp Hb pattern. Our results show that the control of Hb sharpness and positioning, by *hb* self-regulation and Bcd cooperativity, respectively, are separate processes that can be altered independently. Our model, which matches the changes in Hb position and sharpness observed in different experiments, provides a theoretical framework for understanding the data and in particular indicates that spatial bistability can play a central role in threshold-dependent reading mechanisms of positional information.

## Author Summary

Pattern formation during embryonic development, or morphogenesis, is one of the most intriguing problems in biology, entailing the sequence of processes by which a relatively simple system, the fertilized egg, becomes a mature organism. In these processes, the genetic information, stored at the molecular scale in the DNA, is translated into the macroscopic spatial expression patterns that precede the tissue–organ scale of body organization. It can also be understood as a flux of information from the genetic to the organ–system level. In the fruit fly *Drosophila melanogaster*, one of the early processes during its embryonic development is the formation of the sharp Hunchback protein pattern. To generate this pattern, the *hunchback* gene interprets the position-dependent information in the shallow maternal Bicoid gradient and converts it into the sharp Hunchback protein pattern. We propose that bistability in the dynamics of *hunchback* gene regulation can account for this information reading process, and we show that this bistable mechanism can be produced by the ability of this gene to regulate its own expression. The solution of this problem offers new approaches to understand the phenomenon of morphogenesis.

## Introduction

How an embryo achieves pattern and form from an initially undifferentiated state has fascinated people at least since the time of Aristotle. Scientific advances on this began over a century ago, with, for example, the experiments of Hans Driesch on sea urchin embryos [1], from which he proposed that the embryo has a coordinate system specifying cellular position; and from the experiments of Ethel Browne [2], who showed that a piece of hydra mount induced a secondary axis when grafted into the body of another hydra. These and other subsequent results were synthesized by Lewis Wolpert in 1969 [3] into a definition of positional information. According to this concept, the spatial asymmetries of concentration gradients of chemical signals (morphogens) provide positional information during cellular differentiation; each cell (or nucleus) reads its position from the local morphogen concentration and differentiates accordingly. Wolpert's concept of morphogen gradients has become a central tenet of developmental biology [4]–[6]. Modern molecular techniques have demonstrated numerous cases of protein concentration patterns in embryogenesis, and many have been shown to act as morphogens. In the late 1980's, the Bicoid (Bcd) protein gradient was characterized and its concentration-dependent effect on downstream target genes in *Drosophila* was demonstrated [7]–[9]. This has since become one of the most studied examples of morphogen gradient signaling in developmental biology [10],[11].

Reaction-network models have been successfully applied to describe a great variety of systems in physics, chemistry, and biology [12]–[14]. Along with this, many mathematical tools have been developed to support such applications. With these tools, one can show that certain reaction networks may exhibit multiple stationary states, for particular ranges of their rate constants. Bistability is a special case, in which the system can evolve to either of two asymptotically stable steady states (concentration levels). Under certain conditions, spatial patterning or oscillations can arise [15]–[17]. In biology, bistability has long been established in control of the cell cycle and other oscillations [18],[19], and also recently reported in an artificial gene regulation network [20]. In *Drosophila*, spatial bistability has been proposed for dorso-ventral patterning [21],[22].

**Figure 1:**
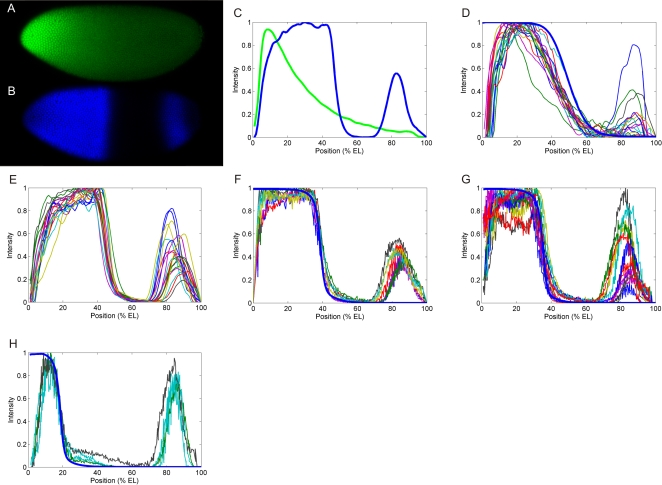
Control of Hb sharpness and position. (A,B) An embryo in mid-nuclear cleavage cycle 14A, with immunostaining to Bicoid (Bcd) (A) and Hunchback (Hb) (B) proteins. Anterior pole on left, dorsal side on top. (C) Fluorescence profiles versus anteroposterior position for A (green) and B (blue). Hb position, 47.1% EL; sharpness, 82.7°. (D) Hb profiles for homozygotes of *hb^14F^*, an allele coding a non-DNA binding Hb protein [52], showing dramatically reduced sharpness (63.9°). Heavy blue line: *hunchback* self-regulatory (HSR) model (Figure 3) prediction for absence of self-regulation. (E) *hb^14F^* heterozygotes and wild-type together, showing similar position (44.3% EL) and sharpness (81.3°). See Figure S2 for non-normalized data. (F) Hb profiles from *bcd^E1^*/+ embryos (Bcd mRNA half-dosage). Heavy blue line: simulation for this background, by reducing Bcd synthesis in the HSR model. (G,H) Hb profiles from embryos expressing one copy of *bcd^K57R^*, an allele affecting Bcd cooperativity [54], gives two outcomes: a small anterior shift ((G); 3.0% change from *bcd^E1^*/+); and a strong anterior shift ((H); 36.7% change from *bcd^E1^*/+). Heavy blue lines: HSR model simulations for weakly and strongly reduced Bcd cooperative binding ((G) and (H), respectively). Maximum intensities are normalized to one, to allow comparison of profile sharpness from different experiments. All embryos are between 26 and 39 minutes into nuclear cleavage cycle 14A, as determined by membrane invagination and the relative position from surface to cortex (see Materials and Methods). In (D) there are two T7 embryos, showing normal posterior pattern (not used for sharpness or position measurements). Individual embryo images are shown in Figure S1.

In early embryogenesis, the diffusion of Bcd protein, translated from mRNA localized at the anterior end of the egg, forms an exponential concentration gradient, establishing the anterior–posterior (AP) axis (Figure 1A and 1C) [8],[23],[24]. Bcd is a transcriptional regulator, and through its asymmetric distribution acts as a morphogen, governing the positions at which the downstream gap genes will be activated. In combination with cross-regulation between these genes, the initial Bcd asymmetry is propagated and refined, establishing the first stage of embryo segmentation [9], [25]–[32]. It is still not well characterized, however, what mechanisms interpret the smooth Bcd positional information into sharp and precisely positioned downstream target gene expression.

*hunchback* (*hb*) is one of the first gap genes activated by Bcd, with strong anterior expression and a sharp on–off boundary at mid-embryo (Figure 1B and 1C) [9], [33]–[35]. Anterior *hb* activation depends on Bcd, as shown by Struhl et al [9] and Driever et al [34], and on its own self-regulation, as already reported by Treisman et al [35] and Margolis et al [36]; many Bcd and Hb binding sites have been identified in the *hb* promoter region, as reported by Treisman et al., among others [35]–[37]. Hb has maternal (*hb_mat_*) and zygotic contributions, and provides positional information for other gap genes, such as *Krüppel* (*Kr*), *knirps* (*kni*), and *giant* (*gt*), and for the homeotic gene *Ultrabithorax* (*Ubx*) [38]–[41]. Removal of both maternal and zygotic *hb* expression results in severe deletions and polarity reversals of the most anterior segments [42]. In normal development, Hb expression drops from highest to lowest over about 10% egg length (Figure 1B and 1C); Considerable attention has been focused on what molecular mechanism generates this Hb sharpness. Early on, it was shown that a *hb* enhancer element of 300 base pairs (bp), containing 6 Bcd binding sites, is sufficient to reproduce the regulatory activity of Bcd on *hb* [9],[34]. It was shown that Bcd binds to these sites cooperatively and it was hypothesized that, due to this cooperativity, a small change in Bcd concentration across some threshold could produce a large change in *hb* promoter occupancy, generating the on–off expression pattern [9], [34], [43]–[46]. However, these studies did not establish that cooperativity is sufficient to generate Hb border sharpness.

To quantify the degree of Bcd's cooperativity, Ma et al. [44] used a six-Bcd site fragment of the *hb* promoter in a DNase I footprint assay, and found a Hill coefficient of about 3.6; Burz et al. [47], using a gel-shift assay with a 230 bp *hb* enhancer, found a Hill coefficient of 3.0. From quantified *in vivo* patterns of Bcd and Hb proteins, Gregor et al. [48], estimated a higher value for this coefficient, of around 5 (though the effects of *hb* self-regulation were neglected, addressed further in the Discussion); and suggested that it could support the proposal of Crauk and Dostatni [49] that Hb expression is entirely determined by Bcd cooperative binding. However, systems with such high Hill coefficients would be expected to show temperature sensitivity. Houchmandzadeh et al. [50] showed that the Bcd gradient is strongly affected by temperature changes of 20°C, but that the Hb pattern is largely unaffected. Dependence on Bcd with Hill coefficients between 3 and 5 would be expected to show far greater effects on Hb than are observed, indicating that Bcd cannot be the only factor regulating the Hb border. The insufficiency of Bcd cooperativity to produce Hb sharpness is also supported by the findings of Simpson-Brose et al. [51], who showed that synergy between Hb and Bcd is necessary to establish the expression patterns of the gap genes, including *hb* itself.

To address these issues, we have taken a combined experimental and theoretical approach to understand how the *hb* gene converts the positional information of the smooth Bcd gradient into a sharp expression pattern. We used wild-type (WT) embryos to experimentally determine how Hb position and sharpness change in time; and we measured how these quantities are affected in embryos mutant for Bcd cooperative binding and for *hb* self-regulation, and by use of an artificial promoter with 6 Bcd and 2 Hb binding sites. We also developed a predictive reaction–diffusion model of *hb* transcription, incorporating both Bcd cooperative binding and *hb* self-regulation. By fitting this model to wild-type Bcd and Hb patterns, we determined kinetic parameters of the model, such as binding constants. With these parameters, our model successfully reproduces the dynamic development of the Hb pattern. By reducing Bcd binding constants or the number of Bcd binding sites, our model reproduces the same mutant phenotypes as our experiments, and predicts a loss of sharpness for a *hb* self-regulation mutant, which we experimentally verified. By applying dynamical systems theory to the model, we show that Hb sharpness is due to spatial bistability stemming from *hb* self-regulation. This means that Hb does not have a single steady-state concentration continuously dependent on Bcd, but that at a threshold Bcd concentration, two stable steady states become available to Hb, and a small change in Bcd concentration can create a dramatic shift in Hb concentration. This shift between steady states is responsible for the sharpness of the Hb boundary. The position of the Bcd threshold is controlled by Bcd cooperative binding, but this mechanism itself is not sufficient to generate *hb*'s expression sharpness.

## Results

### Mutant Genotypes That Affect Hb Sharpening and Positioning

In order to investigate the relative contributions of self-regulation and Bcd cooperativity to Hb sharpness and position, we performed immunohistochemistry assays in wild-type and mutant embryos in nuclear cleavage cycle 14A (Figure 1 and Figure S1; see also Materials and Methods). We used systematic image processing approaches to extract gene expression patterns from confocal microscopy images, to determine embryo ages and to quantify pattern position and sharpness.

**Figure 2:**
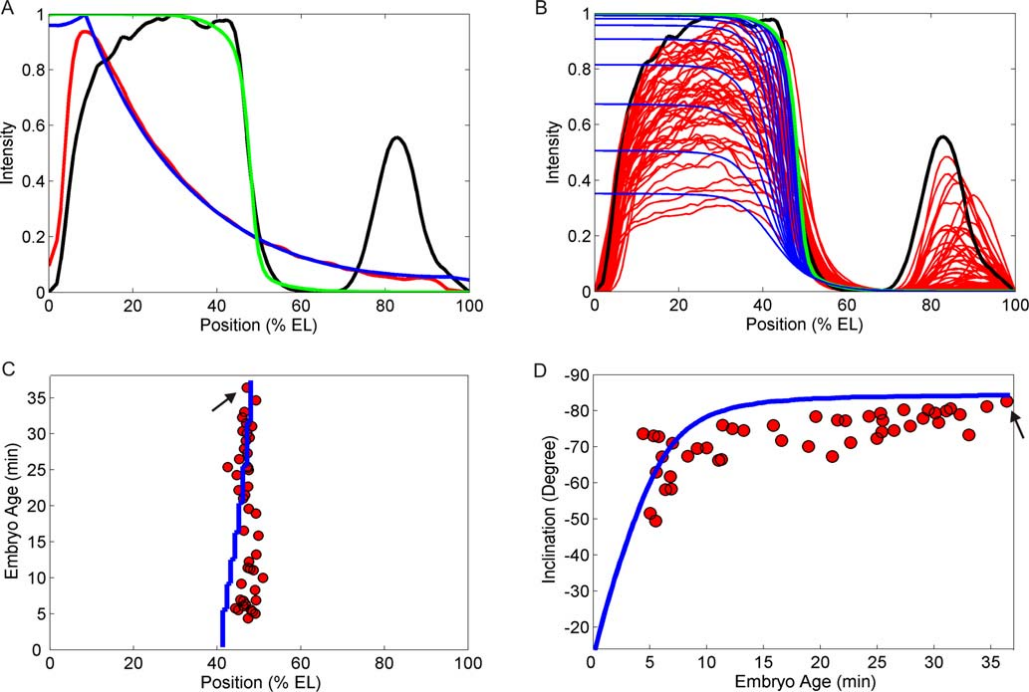
Dynamics of Hunchback (Hb) pattern formation. (A) Fitting the *hunchback* self-regulatory (HSR) model (Figure 3) to the same profiles as in Figure 1C. Red and black lines: experimental Bicoid (Bcd) and Hb, respectively. Blue and green lines: fitting for total Bcd and Hb concentrations (Equations S1.1–2, in Text S1), respectively. (B) 47 Hb wild-type profiles at different ages (red lines). Black line is the oldest one (same as in (A)). Blue lines: Dynamic simulation of HSR model, using kinetic constants determined in (A). Green line is the same as in (A). (C) pattern positions change slightly over time. Red dots and blue line: Hb boundary positions measured from embryos and from simulation in (B), respectively. (D) The pattern quickly achieves mature sharpness, with little change after 10 minutes. Red dots: border inclination (from embryos in (B)) versus embryo age. Blue line: Model prediction for the progressive increase of border inclination, for the simulations in (B). Black arrows indicate the embryo used to fit the model (shown in (A)). See Figure S7 for a direct comparison between data and model time evolution profiles.

The effect of loss of *hb* self-regulation can be directly studied with the loss of function *hb^14F^* allele [52], which codes for a mutant protein having no DNA-binding capacity (i.e., no self-regulation). We scanned 39 embryos expressing this allele and found that embryos homozygous for the *hb^14F^* allele (Figure 1D) have a strong decrease in sharpness (21.8% reduction, from 80.2° in WT to 62.8°), and small shift in position (9.3% decrease, from 47.0% EL in WT to 42.6% EL; Table 1 summarizes sharpness and position for all experiments). The *hb^14F^* homozygotes were easily identified by low signal intensities [53] (see Figure S2 for non-normalized profiles). Heterozygote (*hb* ^14F^/+) and WT embryos were not easily distinguished, and are shown together in Figure 1E. The means for this group show little or no change from WT: sharpness shows a 1.3% change (from 80.2° to 81.3°), and position shows a small shift, from 47.0% EL to 44.3% EL (5.7% change; Figure 1E). In order to compare Hb patterns between different assays, in which absolute intensity varies, all experimental and theoretical profiles in Figure 1 are normalized. With this, our measure of sharpness is determined by the AP projection of the profile (see Figure S3); this is covered in more detail in the Discussion. Note that all *hb^14F^* homozygote embryos display lower sharpness than any WT embryo older than 8 minutes in cycle 14 (the timing of sharpness maturation is discussed further in Figure 2 and in the Discussion).

**Table 1:** Mean positions and sharpness (inclination) for all embryos and simulations presented.

Embryos Background/Simulation	Mean Position (% EL)	Position Std. Dev.	Mean Inclination (degrees)	Inclination Std. Dev.
WT Hb pattern used for fitting	47.1	NA	82.7	NA
HSR Model (6B2H) Fit to WT	48.0	NA	84.3	NA
WT *hb*	47.0	1.5	80.2	1.7
*hb* ^14F^ heterozygotes and WT	44.3	1.8	81.3	1.3
*hb* ^14F^ homozygotes	41.9	3.3	63.9	3.9
HSR model (6B0H) for *hb* ^14F^ homozygotes	49.0	NA	68.3	NA
*bcd* heterozygous, *bcd* ^E1^/+	39.2	0.2	80.6	1.3
HSR (6B2H) model for *bcd* heterozygotes.	40.1	NA	83.4	NA
*bcd* ^K57R^ weak	37.6	2.3	80.6	1.3
HSR model (6B2H) for *bcd* ^K57R^ weak	38.0	NA	83.8	NA
*bcd* ^K57R^ strong	24.8	2.9	82.2	1.2
HSR model (6B2H) for *bcd* ^K57R^ strong	24.4	NA	84.3	NA
pThb5 lacZ (FISH)	45.7	1.8	72.7	0.9
pThb5 lacZ (*in situ hybridization*)	47.3	2.8	74.9	2.4
HSR model for pThb5 (6B2H_lacZ)	48.0	NA	74.9	NA
WT *hb* RNA (simultaneous FISH Immunofluorescence)	45.0	1.0	84.2	1.4
WT Hb protein (simultaneous FISH Immunofluorescence)	45.4	1.0	82.5	1.1

All embryos are between 26 and 39 minutes into nuclear cleavage cycle 14A. NA means not applicable.

Despite Bcd's role in AP positioning during early *Drosophila* development, *bcd* heterozygotes (*bcd^E1^/+*) are highly viable embryos, in which, for example, among 593 embryos only 4% were unhatched, and no larval head defects were found [46]. In these mutants, we found that Hb was shifted anteriorly (16.6% change, from 47.0% EL in WT to 39.2% EL), as previously reported [8],[50], but sharpness was not affected (0.5% change; from 80.2° in WT to 80.6°; Figure 1F).

Mutant *bcd* genes encoding proteins specifically defective in cooperative DNA binding have been isolated by Hanes and collaborators [46],[54], using a genetic screen in yeast. It was shown that these mutations do not disrupt the DNA recognition or transcriptional activity of Bcd. We used one of these mutants, *bcd^K57R^*, to analyze the effect of Bcd cooperative binding on Hb sharpness and position. In embryos with one dosage of the *bcd^K57R^* allele (in a null Bcd background), incomplete penetrance gives two outcomes: embryos with a weak response to the Bcd defect (Figure 1G; showing a small anterior shift compared to *bcd^E1^/+*, from 39.2% EL to 37.6% EL); and embryos with a strong response, which have a large anterior shift compared to *bcd^E1^/+* (Figure 1H; from 39.2% EL to 24.8% EL). In both cases, sharpness is not reduced (0.5% and 2.5% change from WT, for weak and strong mutants, respectively).

Driever et al. [43] used a series of lacZ constructs to describe the effect of high and low affinity binding sites for the establishment of the localized zygotic expression domains. These constructs have a lacZ coding sequence attached to different fragments of the *hb* promoter sequence. To make flies carrying these constructs, they are introduced into the fly genome, but the WT background is maintained. In one of these constructs, pThb5, the promoter is a portion of the native sequence, having 6 Bcd and 2 Hb binding sites (see Figure S4). These constructs do not show self-regulation, since the protein encoded by them (β-galactosidase) has no transcriptional activity. pThb5 expression has significantly reduced sharpness (from 80.2° in WT to 72.7°, Table 1; see Figure S5 and Figure S6 for lacZ expression patterns) and a slight anterior shift in position (from 47.0% in WT to 45.7% EL). In addition, we found that pThb5 expression is sharper than *hb* ^14F^ (72.7° vs. 63.9°), indicating that Hb protein, normally expressed in these flies, can increase the sharpness of pThb5 expression (This effect of the Hb WT expression was predicted by our model, as shown below.)

### A Reaction–Diffusion Network Model Reproduces the Dynamics of Hb Pattern Formation

**Figure 3:**
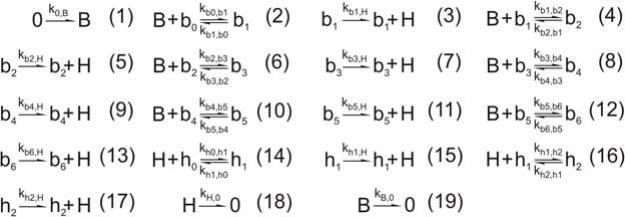
The *hunchback* (*hb*) self-regulatory (HSR) model to simulate *hb* transcriptional activation by Bicoid (Bcd) and self-regulation. (1): Bcd (B) synthesis from a source term; (2*n*, *n* = 1, …, 6): Bcd binding to *hb* promoter; (14, 16): Hb (H) binding to *hb* promoter; (1+2*n*, *n* = 1, …, 6) and (15, 17): Hb synthesis; (18 and 19): Hb and Bcd decay, respectively. b_n_ and h_n_ represent the fragments of *hb* promoters containing 6 Bcd and 2 Hb sites, respectively; subscripts *n* indicate how many Bcd or Hb molecules are bound. 0 denotes either inert or constant concentration species (e.g. mRNA). k_b0,b1_ indicates the transition from b_0_ to b_1_ states; k_b1,H_ indicates production of H from b_1_, and so on. We introduced cooperativity by taking k_b(n-1),bn_ = factor^n^.k_b0,b1_ and k_bn,b(n-1)_ = k_b1,b0_ for *n* = 2, …, 6. In addition, we set k_bn,H_ = (1+Synt_Factor).k_b(n-1),H_ for *n* = 2, …, 6 to account for the effect of multiple protein binding to the gene promoter. These relations strongly reduced the number of parameters, and the model fitting was not sensitive to changes in them.

To investigate what causes the changes in positioning and sharpness shown in Figure 1 and Table 1, we developed a predictive reaction–diffusion model. This *hunchback* self-regulatory (HSR) model captures both Bcd cooperative binding and *hb* self-regulation, with six Bcd sites and two Hb sites driving *hb* expression. This model readily reproduced the phenotypes of the WT and mutant experiments, allowing us to make predictions and gain new understanding of the molecular mechanisms producing the measured macroscopic patterns. The model reactions are summarized in Figure 3. *hb* expression requires two steps: Bcd and Hb protein binding to *hb* promoter (reversible reactions (2*n*, *n* = 1, …, 6) and (14, 16), respectively); and Hb protein synthesis (irreversible reactions (1+2*n*, *n* = 1, …, 8)). Reaction (1) represents Bcd production; reactions (18) and (19) represent Bcd and Hb degradation, respectively.

We used Fick's Law to describe Bcd and Hb diffusion, and the Law of Mass Action [55] for the reactions in Figure 3, to derive a system of coupled partial differential equations (PDE's; see Text S1) for the species *B*, *H*, *b_0_–b_6_*, *h_0_–h_2_* (only species B and H are allowed to diffuse). The dynamics of the system are completely described by initial conditions, diffusion coefficients and the kinetic constants, found by fitting model output to experimentally measured expression patterns.

We used a finite difference method to solve the model PDE's (see Text S1) and a steepest descent method to determine the *k* parameters, by fitting the total Bcd ([*B*]_T_) and Hb ([*H*]_T_) concentrations (Text S1, Equations S1 and S2) to the respective patterns of an embryo in mid-nuclear cleavage cycle 14 (about 36.4 minutes into the cycle, Figure 2A; see Materials and Methods for age determination method). The Bcd gradient was fit first, by using a zero initial concentration and fitting the model to Bcd experimental data (Figure 2A) without the Hb reactions (Figure 3, reactions 3, 5, 7, 9, 11, and 13–18), and with Bcd production (Figure 3, reaction 1) only at 9% EL (the position of maximum Bcd level in the data). The Hb reactions were then taken into account, and the model fit to the Hb experimental data (Figure 2A). With the *k*'s determined (Table S1), we simulated the dynamics of the HSR model (Figure 2B), using zero initial Hb concentration (embryos lacking maternal Hb develop normally [42]; also, final concentrations are largely unaffected by low initial [H_T_]—see Discussion). The HSR model qualitatively reproduced the time development of the Hb pattern (Figure 2B, 59 embryos of different ages), even though it was fit to only one pair of Bcd-Hb patterns (Figure 2A; see Figure S7 for a direct comparison between data and model time evolution profiles). The match was best with respect to sharpness increase (Figure 2D); for position, the model shifted more than observed (Figure 2C; see also Figure S7A and S7B), possibly reflecting the simplified aspect of the model, like the number of Hb or Bcd sites. Figure 2 indicates that developmental age, after the transient behavior in the first 10 minutes of cycle 14, is not a significant factor in sharpness or position.

### Spatial Bistability, Produced by *hb* Self-Regulation, is Responsible for the Sharp Hb Border

**Figure 4:**
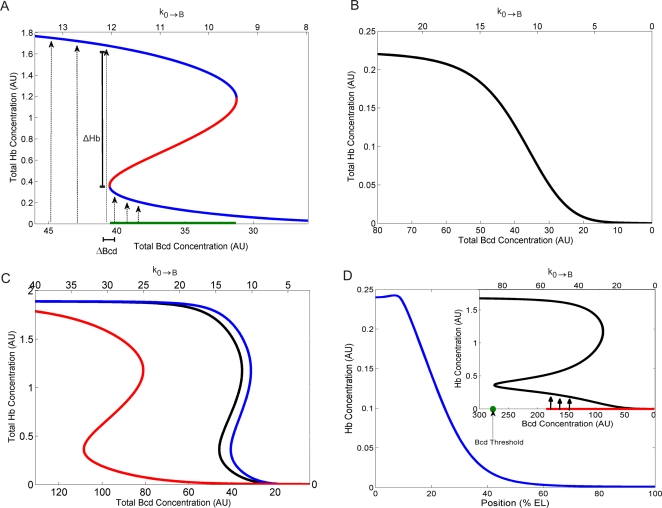
Bifurcation diagrams and simulations for the *hunchback* (*hb*) self-regulatory (HSR) model. (A) Bistability: solid blue curves are the stable steady states for Hb total concentration [H]_T_ (y-axis) for a given Bicoid (Bcd) total concentration [B]_T_ (bottom axis), or as Bcd production rate (k_0,B_, top axis). Red curve is unstable steady states ([H]_T_ will evolve away from such concentrations). Hb concentration will evolve to the low steady states (short dashed lines) at low Bcd concentration, but abruptly steps up to the high steady states (long dashed lines) as Bcd concentration, [B]_T_, moves out of the bistable region (31.2<[B]_T_<40.5; green line on bottom axis). (B) Loss of bistability in the absence of self-regulation: bifurcation diagram for HSR model without self-regulation reactions (14–17) shows no bistability. (C) Reduction in Bcd cooperative binding shifts bistable region toward high [B]_T_ regions. Black and Red: bifurcation diagrams for heavy blue lines in Figure 1G and 1H, respectively (simulations for reduced Bcd cooperative binding (Figure 3; Table S2); blue line, wild-type diagram (same as in (A)); (D) Simulation for reduced number of Bcd binding sites (removing reactions 10–13) shows disrupted sharpness since Bcd concentrations (red line in the bifurcation diagram, small box) do not reach the bistable threshold (green dot).

Once we established that the HSR model accounts for wild-type expression, we could analyze its dynamics to determine what is responsible for converting the smooth Bcd spatial distribution into the sharp Hb pattern. We chose Zero Eigenvalue Analysis [56]–[59] as a technique for searching parameter values that produce bistability in our model. This method establishes a set of restrictions (the sign compatible relations, see Text S2) which must be met in order for bistability to occur. Using this technique on a simplified HSR model [60], with reduced number of Bcd binding sites (reactions 4 to 13 removed, Figure 3) and normal Hb binding, we demonstrated that the model does have bistable solutions (see Text S2). Although this analysis was performed for a well-mixed system, where concentrations are assumed to be uniform, the bistable behavior is also found in numerical solutions of the full model, where spatial distributions of concentrations are considered. The bifurcation diagram (Figure 4A), plotting [*H*]_T_ steady-state solutions for various k_0,B_ and [*B*]_T_, shows that for Bcd concentrations within the bistable region (green line), Hb concentration has two stable solutions (blue lines), separated by an unstable solution (red line). This bistability causes a very large change in Hb concentration (ΔHb, from one stable branch to the other) over a small change in Bcd concentration (ΔBcd) as it passes through a threshold (at the anterior boundary of the bistable region). In the *Drosophila* embryo, the Bcd gradient provides different concentrations along the anterior–posterior axis, which work like different initial conditions in the well-mixed system. It creates a sharp Hb border (Figure 1C) at the position where Bcd crosses this threshold. Comparison between *hb* RNA and protein profiles shows that RNA patterns are sharper than protein ones (84.2° and 82.5° mean sharpness, respectively; Figure S8 and Table 1). These findings combine with our results above, to indicate that Hb sharpness results from spatially bistable dynamics, which is due to *hb* self-regulation. In the HSR model, if the *hb* self-regulation reactions (Figure 3, reactions 14–17) are removed, the network loses bistability and has only a single steady state (Figure 4B), in which Hb varies smoothly with Bcd.

With Hb bistability characterized in the model, we can proceed to simulating the macroscopic behaviors (i.e., expression phenotypes) shown in Figure 1, by altering binding strengths and site numbers, to reproduce the corresponding mutant genotype. For example, to model *hb* expression in the *bcd* ^E1^/+ heterozygotes we reduced the Bcd source term (k_0,B_) to 66.6% of original (see Table S2), finding anteriorly shifted Hb pattern (16.5% change from WT simulation) without disrupting sharpness (1.0% change from WT), in agreement with data (Figure 1F, heavy blue curve; Table 1). Hb sharpness was maintained because reduction of Bcd production by this amount does not change the model's bistable phase portrait, Figure 4A, but does shift the position of the Bcd threshold anteriorly. To simulate loss of self-regulation, we removed reactions 14–17 (Figure 3) from the full model (6B2H, for 6 Bcd and 2 Hb sites), to give 6B0H sites. The model predicts a loss of sharpness (19.0% change from WT; from 84.3° to 68.3°; Figure 1D, heavy blue curve, Table 1) in qualitative agreement with *hb^14F^* homozygote experimental data (which showed a 20.3% change from WT). The bifurcation diagram for 6B0H sites (Figure 4B) shows that this loss of Hb sharpness is due to loss of bistability, since Hb concentration becomes a smoothly decreasing function of Bcd concentration.

The pThb5 construct contains an estimated six active Bcd sites and two Hb sites (Figure S4), but it does not exhibit self-regulation because the protein coded by it has no transcriptional activity. To reproduce the lacZ expression of pThb5 we derived an extra set of reactions by replacing H with L (LacZ) in reactions (1+2*n*, *n* = 1, …, 8; and 18), and added these reactions to the full model; with this model (6B2H_lacZ) we have no self-activation for lacZ, but we still take into account both Hb sites in the lacZ promoter. We found no shift in position, but did find an 11.1% loss of sharpness from the WT simulation (Table 1, Figure S3), in agreement with the experimental loss of sharpness from WT to lacZ (9.3%). This indicates that the loss of sharpness for pThb5 is caused by the lack of self-regulation. The lacZ experimental patterns are sharper than *hb* ^14F^ (72.7° and 63.9°, respectively), probably due to the Hb sites in the construct promoter region. Our model predicts this effect, with sharpness for 6B2H_lacZ (74.9°) higher than sharpness for 6B0H (68.3°).

### Bcd Controls Hb Position, not Sharpness

The loss of sharpness for *hb^14F^* homozygotes, Figure 1D and Table 1, demonstrates that cooperative Bcd binding is not sufficient to generate the sharp Hb border, since Bcd cooperativity is not affected in this *hb* allele. Bcd cooperative binding does play an important role in Hb pattern positioning, however, as demonstrated by the *bcd^K57R^* results (Figure 1G and 1H; Table 1; see also [43]), in which pattern position is altered without affecting sharpness. We simulated the reduced cooperativity in *bcd^K57R^* embryos [54] by reducing the Bcd binding constants in reactions (2*n*, *n* = 1, …, 6) by dropping *factor* to 67% and 95% of original, to simulate the strong and weak mutants, respectively (Figure 3 caption, Table S2). Figure 4C shows that this reduction in cooperativity shifts the bistable region towards regions of high Bcd concentration (Figure 4C, red and black lines), anteriorly shifting the pattern without disrupting its sharpness, in agreement with the data (Figure 1G, heavy blue line; Figure 1H, heavy blue line; Table 1). These results show that Bcd cooperative binding controls the position at which the Hb bistable switch occurs.

Though not sufficient for sharpness, Bcd cooperativity is necessary for Hb bistability to produce sharpness. We can demonstrate this by simulating a strong decrease in cooperativity *in silico* by a decrease in the number of Bcd binding sites (removing reactions 10–13, giving 4B2H sites) without affecting self-regulation. These simulations show a strong reduction in *hb* activation, giving both a strong anterior shift and a drop in sharpness (Figure 4D). The small box in Figure 4D shows that bistability was not disrupted, since self-regulation was not affected, but the Bcd threshold was shifted to a very high concentration not reached by the Bcd gradient (indicated by red line). This result indicates that Bcd cooperative binding is necessary for *hb* activation to reach its bistable threshold, which in turn is necessary for sharpness to occur.

The above results show that small disruptions of Bcd cooperative binding result in positional shifts, without loss of sharpness, while large enough disruptions of cooperative binding also disrupt Hb sharpness, since the bistable switch is not reached. However, the bistable switch itself can only be produced by *hb* self-regulation.

## Discussion

*hb* is one of the most studied developmental genes in *Drosophila melanogaster*. Since its discovery, many aspects of its transcriptional regulation and roles in establishing the segmented body plan of the embryo have been studied. We have shown, using a predictive reaction–diffusion model as well as a series of experimental data, that *hb* reads out the positional information of the morphogenetic Bcd gradient with bistable kinetics, resulting from *hb* self-regulation. The sharp Hb border is generated by the transition from one stable Hb concentration to the other, and the AP position where this switch occurs is determined by a threshold in the Bcd concentration, which establishes the *hb* activation level through cooperative binding. Our model reproduces the time development of the sharp border formation and predicts that if *hb* self-regulation is removed, bistability will be lost and sharpness disrupted. The expression pattern of the *hb* self-regulation mutant, *hb^14F^*, confirms this prediction. Our analysis confirms earlier observations of the role of Bcd cooperative binding in positioning, but establishes that this mechanism is not sufficient for sharpness. Here, we discuss these conclusions in more detail in the context of the literature.

### Bcd Cooperativity

The role of Bcd cooperative binding on Hb positioning has been demonstrated since 1989 [9],[34],[43]. For example, Driever et al. [43] used a selection of artificial lacZ constructs, each containing some portion of the native *hb* regulatory sequence, with varying numbers of high and low affinity Bcd binding sites. They showed that reducing the number or strength of Bcd binding sites shifted the lacZ expression anteriorly, demonstrating the role of cooperativity for pattern positioning. Increasing the binding strength or number of sites gave posterior shifts and sharper borders, suggesting that cooperativity could also be responsible for Hb sharpness. However, even for the construct with the highest number of sites (12; 6 strong and 6 weak), which showed the strongest expression level, pattern was not as sharp as wild-type Hb. While a role for cooperative binding in sharpening was suggested by these results, the authors noted this could not be firmly concluded from their data [43]. Struhl at al. [9] also observed shallower than endogenous Hb borders with a series of similar lacZ constructs. Based on their results, Driever et al. [43] proposed a ‘gradient-affinity model’, wherein target genes with high affinity binding sites, like *hb*, would be efficiently expressed even at low Bcd concentrations, and target genes containing low affinity binding sites would be positioned in more anterior positions.

Our model reproduces these positioning effects of Bcd cooperative binding, as shown in the simulations in which binding site strength was varied (e.g., Figure 1F–H). It also explains that the border of lacZ expression patterns is not as sharp as wild-type Hb because of the loss of self-regulation in such constructs (Figure S5 and Figure S6). These results indicate that bistability can play a role in the gradient-affinity model, since they show that changing the cooperativity level shifts the Hb pattern (Figure 1G and 1H) but does not change its sharpness, allowing an on–off expression boundary to be placed at many positions in the embryo.

Instead of changing cooperativity by changing the binding sites in artificial constructs, Burz and Hanes [54] generated several Bcd cooperativity mutants, such as the *bcd* ^K57R^ used in this study. They showed that this mutant is stable *in vivo* (in yeast cells) and is not affected in its DNA recognition, nuclear entry, or transcriptional activity characteristics [46],[54], and *in situ* hybridization showed that localization and expression of *bcd^K57R^* mRNA is normal [46]. Through analyzing the expression pattern of the gap genes *hb*, *gt* and *Kr* in this mutant, Lebrecht et al. [46] showed that cooperative Bcd binding is critical for embryonic patterning. That study also reported a reduction in Hb sharpness, contrary to what we report here (see Figure 1G and 1H and Table 1). In [46], sharpness (slope) was calculated on non-normalized data, which makes their results susceptible to artificial variations in gene expression levels that can occur at many steps in a staining assay, such as embryo fixation and permeabilization. Using non-normalized data and measuring the slope by the quotient between profile intensity, Δ*y*, by the distance along AP axis, Δ*x*, introduces variability in slope due to variability in Δ*y*, see Figure S3. Here, we present new results for Hb sharpness, computing slope with normalized data (Δ*y* = 1) to reduce the contribution of these experimental errors and to compare mutants with different levels of expression. Our method, similar to that used by Crauk and Dostatni [49], depends only on how far it takes Hb to drop from ‘on’ to ‘off’ expression (Δ*x*). Recently, Gregor et al. [61] have shown that immunofluorescent signals are proportional to protein concentration plus a nonspecific background. This indicates that normalizing immunofluorescent signals provides an equivalent result to normalizing real concentrations. With this approach, we do not find a significant difference in sharpness between *bcd* ^K57R^, *bcd* ^WT^, or *bcd* ^E1^/+.

Gregor et al. [48] recently presented quantitative data comparing Bcd and Hb intensities from whole embryos, and analyzed the precision of this input/output relation. They fit the Hill equation (Equation 1) to the Bcd/Hb input/output relation and estimated that Bcd binds to the *hb* promoter with a Hill coefficient of 5; somewhat higher than the in vitro values [44],[47], but within the known number of Bcd binding sites [9],[34]. However, they neglected the contribution of *hb* self-regulation in establishing the levels of Hb protein, so the value reported for Bcd_1/2_ would not produce half-maximal Hb synthesis in the absence of self-regulation (see Text S3 for more details). In other words, to reach maximum Hb production without self-regulation would require a higher Bcd concentration, and a higher value for Bcd_1/2_. Calling this corrected value Bcd_1/2_^Cor^, Bcd_1/2_^Cor^>Bcd_1/2_, which means that ln(Bcd/Bcd_1/2_^Cor^)<ln(Bcd/Bcd_1/2_), and using Equations 3 and 4 shows that *n* ^Cor^>*n* (see Text S3 for derivation of these equations). This indicates that a corrected Hill coefficient (*n* ^Cor^) should be higher than that reported by Gregor at al. and likely higher than the six Bcd binding sites known for *hb* regulation [9],[34], making a claim that Bcd cooperativity determines Hb sharpness unlikely. In [48] it was argued that the effect of additional factors, uncorrelated with Bcd, would require *hb* readout of Bcd concentration to be even more reliable than reported. However, this argument does not justify neglecting *hb* self-regulation for establishing levels of Hb protein, because Hb precision (measured by standard deviation) is not correlated with Hb protein levels, as shown in [48]. In other words, *hb* self-regulation may not influence the precision of the readout process, but it does determine protein levels, and this role cannot be neglected in calculating the Hill coefficient.
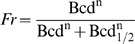
(1)

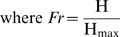
(2)

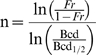
(3)

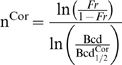
(4)


### The Requirement of Bistability for Sharpness

Our HSR model, though fit to WT data, predicts the loss of sharpness we found experimentally in the self-regulatory mutant, *hb* ^14F^ (Figure 1D and Figure S1). The experimental results directly support the need for *hb* self-regulation for sharp pattern development. *hb* ^14F^ is a lack of function mutant, generated by Lehmann et al. [42], which forms normal mRNA [51] but has a truncated protein with no DNA binding capacity. The protein is stable, persisting into central nervous system development [62], and has been visualized with Hb antibody [53] staining at lower intensity than WT (Figure S2; we measured intensity at 10–20% WT, comparable to the 10% reported in [53]). Since the Bcd protein or its binding are not affected in these mutants, these embryos clearly show that Bcd cooperative binding is not sufficient for producing Hb sharpness. Our results with *hb* ^14F^ agree with the observations of Houchmandzadeh et al. [50] that expression in the *hb^6N^* allele (also with non-functional protein) suggested a role for self-regulation in sharpening. Our model shows that loss of self-regulation disrupts the bistable behavior in *hb* ^14F^ expression, resulting in the loss of sharpness. Similarly, expression of the pThb5 lacZ construct shows reduced sharpness in comparison to WT (Figure S5, Figure S6, and Table 1), since the protein coded by lacZ (β-galactosidase) is not self-regulatory. Construct sharpness is greater than *hb* ^14F^ sharpness, however. The model predicts this, by taking into account that native, patterned Hb protein can bind in the construct promoter (compare Figure 1D to Figure S5 and Figure S6; see also Table 1 and Figure S4).

Crauk and Dostatni [49] recently reported sharp expression for a lacZ construct containing only three (strong) Bcd binding sites. We found lowered sharpness for the pThb5 construct (Figure 1H), which contains six Bcd binding sites (three strong, three weak; as well as two Hb and Kr sites). Since this is opposite to what any cooperative effect should be for increasing sites, the differences are likely to be methodological. We used both whole mount fluorescent *in situ* hybridization (FISH; Figure S5) and traditional in situ hybridization (Figure S6; as used in [49]) to visualize lacZ expression. Both methods gave similar measures of sharpness (Table 1), but the enzymatic staining is more susceptible to signal saturation and tends not to be proportional to RNA concentration. Crauk and Dostatni [49] also reported reduced sharpness in embryos with truncated Bcd proteins, *Bcd-ΔC* and *Bcd-ΔQC* (with specific defects in protein activity). In light of the *hb* transcriptional dynamics found in our analysis, we believe such alterations to Bcd could cause transcription to remain sub-threshold for bistable activation, similar to Figure 4D.

### The Method Used for Embryo Staging

All embryos used in Figure 1 and Table 1 were in nuclear cleavage cycle 14A (precellular blastoderm), within time classes T5 to T6 (26 to 39 min into this cycle), during which Hb levels are at their highest (Figure 1D shows two T7 embryos, to show the normal posterior patterning in these mutants). We staged each embryo by established methods [63], following dorsal membrane invagination measured from images obtained by Differential Interference Contrast (DIC) optics. For comparing Hb dynamics and model simulation (Figure 2), we used embryos in the first 36.4 minutes of cycle 14A. While Hb expression dynamically amplifies over this period (Figure 2B), the mature sharpness is reached within 5–10 minutes, after which it is stable (Figure 2D). Note that Figure 2D shows that Hb pattern in all WT embryos older than 8 min in cycle 14 are sharper than any embryo in Figure 1D.

### Maternal Hb

The earliest Hb protein pattern in the embryo is of maternal origin. Before nuclear cleavage cycle 8, maternal *hb* mRNA is distributed uniformly throughout the egg, but its translation is repressed by the posterior Nanos (Nos) protein gradient, resulting in a smooth anterior gradient of maternal Hb (Hb_mat_) protein. This is gradually substituted by the zygotically expressed Hb, starting in cycle 11 [27], [33], [64]–[69]. To see the effect of these early Hb distributions on cycle 14 dynamics, we ran HSR model simulations with an initial Hb pattern taken from cycle 13 data (Figure S9; parameters and data from same assay as in Figures 1C and 2A). Simulation results were the same as in Figure 2, indicating no effect from the Hb initial condition. This behavior is related to the bistable behavior of Hb: the diagram in Figure S10 shows that, inside the bistable region, only relatively high initial conditions (above the unstable branch) can produce high Hb concentrations. It indicates that the initial concentrations of Hb, determined by zygotic production, are low and not sufficient to carry the system through the transition from lower to upper stable branch. This agrees with previous results showing that embryos lacking maternal Hb develop normally [42],[65],[66]. Like the *Drosophila* embryos, the HSR model is robust to variability in Hb initial concentration.

### The Method of Constructing Reaction-Network Models

The method we have used to construct the HSR network, describing *hb* regulation by Bcd cooperative binding and *hb* self-regulation, can be readily applied to other genetic regulatory systems in *Drosophila* or other organisms, since the regulatory interactions are general. We avoided using a Hill kinetics approach to model cooperativity because this would require some questionable assumptions, such as all six sites being equal, which is counter to published Bcd binding data [43],[47], and bound simultaneously, which is highly improbable. One advantage of using the Hill equation could be its few number of parameters; however, using relations k_b(n-1),bn_ = *factor* ^n^.k_b0,b1_ and k_bn,b(n-1)_ = k_b1,b0_ for *n* = 2, …, 6 in reactions (2+2*n*, *n* = 1, …, 5) allowed us to describe cooperativity with just three parameters, k_b0,b1_, k_b1,b0_ and *factor*. The effects of more or less binding sites and more or less transcriptional regulators can easily be built into our kind of model. Our method allows for a direct link between macroscopic pattern formation and its molecular basis. As well, such a model is amenable to mathematical analysis with modern nonlinear techniques, which have developed rapidly in recent years [17], [55], [70]–[73]. In the present example, using such techniques to search for multiple steady states allowed us to identify the bistability inherent in the self-regulatory reactions, and determine the model parameters necessary for triggering this.

### The Bistable Behavior

In reaction networks, bistability is frequently verified by changing the initial concentration of one species, in the well-mixed system, and checking the concentrations of all other species when the system reaches the stationary state. In a monostable regime, small variations in the initial concentration generally produce small variations in the stationary state. However, if the concentration is in the vicinity of a threshold, where the transition from the monostable to the bistable regimes occurs, small changes in the initial condition can produce large variation in the stationary state, because the concentrations of the species can follow a completely different trajectory (i.e., sequence of intermediate concentrations), ending up in a very different stationary state (Figure 4A). In our spatially-patterned case, the anterior–posterior Bcd gradient provides many different concentrations that work like different initial conditions in the well-mixed system; in such a way that at the position where Bcd crosses the threshold (Figure 4A), the Hb stationary concentration changes abruptly, producing the sharp Hb border (Figure 2A).

The origin of bistability in the Bcd-Hb system is a consequence of the positive feedback of *hb* self-regulation. If Hb production is not high enough, self-regulation can only produce a small change in Hb production, and the consequent increase in degradation counteracts almost all increase in Hb production; this regime occurs in the posterior half of the embryo. If Hb production is more effectively increased, by increasing Bcd concentration, the positive feedback can produce a certain additional amount of Hb protein, which can be sufficient to start increasing Hb production more efficiently. If this occurs, the additional amount of Hb will increase the feedback even more strongly, ending up in a completely different regime, having higher Hb concentrations; this regime occurs in the anterior half of the embryo.

### Concluding Remarks

Our data and model show that positioning and sharpness of the Hb pattern are separable processes. With the *hb^14F^* allele and the pThb5 construct, we show that sharpness can be disrupted with self-regulation defects; and our theoretical analysis suggests this is due to loss of bistability. Earlier work has suggested many of the shifting and sharpening effects we find here. However, there has been debate about the relative roles of the transcriptional regulators: some studies have suggested a role for *hb* self-regulation in sharpening [50], while others indicate that it could be completely controlled by Bcd [48],[49]. It has also been known that the number of Bcd binding sites in the *hb* promoter affects pattern position [9],[34],[43]. Our data and model offer a synthesis: positioning is largely dependent on the Bcd occupation states of the *hb* promoter, but sharpening is a result of bistability in the *hb* activation dynamics, caused by *hb* self-regulation. Bcd cooperativity, through affecting *hb* transcription, determines the threshold at which bistability occurs, but is not itself sufficient for sharpening.

In 1977, Lewis at al. [74] used theoretical arguments to suggest that bistable control can account for the interpretation of gradients in positional information. More recently, bistability has been found in many complex biological processes [14]–[16], [18]–[21],[75] and spatial bistability has been proposed in dorso-ventral patterning in *Drosophila* [21],[22]. Here, we have combined experiments, modeling and analysis to suggest that this dynamic feature may also be central to AP patterning, and that for *hb* transcription bistability arises from the convergence of two regulatory mechanisms (Bcd cooperative binding and *hb* self-regulation). This provides a specific mechanism to the earlier indication that Bcd and Hb synergy is required for *Drosophila* gap patterning [51]. Moreover, in agreement with Lewis et al. [74], our findings indicate that bistability may be central to threshold-dependent reading mechanisms of the positional information established by smooth maternal signals.

Our approach, of developing a kinetic transcriptional model from molecular data such as binding sites and regulatory interactions (repression or activation), using dynamical systems theory to determine the model dynamics, and confirming the model predictions against quantitative experiments, could be used for uncovering regulatory mechanisms in many other pattern formation systems, in fruit flies and in other organisms.

## Materials and Methods

### Dataset

We stained for Bcd and Hb proteins in WT Oregon-R embryos, as well as in the *hb* mutant *hb^14F^* [52], and two *bcd* mutants [46] (*bcd^K57R^*, *bcd^E1^/+*). lacZ expression for the pThb5 construct (driven by a fragment of the *hb* promoter; [43]) was visualized by two methods for staining β-galactosidase mRNA (Table 2). The simultaneous Hb protein and RNA visualization was also done in WT Oregon-R embryos.

**Table 2:** Methods for obtaining expression patterns, for the specified numbers of WT and mutant embryos.

Method	Genes					
	*hb* ^WT^ *bcd* ^WT^	*hb* ^14F^ *bcd* ^WT^	*hb* ^WT^ *bcd* ^K57R^	*hb* ^WT^ *bcd* ^E1^/+	*lacZ*	*hb* ^WT^ *RNA hb* ^WT^ *prot.*
FISH^a^	–	–	–	–	9	13
Immunofluorescence^b^	50	39	23	7	d	13
Enzymatic staining^c^	–	–	–	–	21	d

a Whole mount fluorescent in situ hybridization.

b Fluorescence immunostaining.

c Whole mount in situ hybridization with digoxygenin-labeled RNA.

d All lacZ embryos were co-stained for Bcd and Hb proteins.

### Preparation and Staining

As outlined in Table 2, three different staining procedures were used for obtaining expression patterns. For all procedures, embryos were dechorionated; heat fixed in NaCl 0.7%+Triton-X100 0.05% for 3 seconds and immediately chilled in ice; and devitellinized with methanol.

For protein staining [76], embryos were incubated with guinea pig and rat primary antibodies to Hb and Bcd, respectively, followed by secondary antibodies labeled with Alexa Fluor 647 (to Hb) and 488 (to Bcd; Molecular Probes). All antibody incubations and washes were done in PBS+0.1% Tween-20. Blocking was done in Western Blocking Reagent (Roche), diluted 5 times. All secondary antibodies were preabsorbed by incubating them with 0- to 12-h-old WT embryos for at least 2 h at 4 C.

For the lacZ embryos, we used simultaneous immunostaining to Hb and Bcd and in situ hybridization. With FISH, we followed the method of Janssens et al. [76]: a lacZ riboprobe was prepared with a 2.5-kb PvuII lacZ fragment blunt-cloned into the EcoRV site of pBluescriptIIKS+ (gift from S. Small), labeled with fluorescein by transcription using T3 polymerase. After hybridization, lacZ mRNA was visualized by sequential incubation with rabbit antibody to fluorescein (Molecular Probes), followed by antibody to rabbit labeled with Alexa Fluor 488 (Molecular Probes). The embryos were simultaneously stained for Hb and Bcd proteins, as in the previous paragraph, using secondary antibody labeled with Alexa Fluor 555 to detected Bcd. Alternately (Figure S6), some lacZ embryos were measured via enzymatic staining (whole mount in situ hybridization): β-galactosidase mRNA was hybridized in situ with a digoxygenin-labeled DNA probe, following standard protocols [67]. The hybridization products were detected with a phosphatase-coupled antibody against digoxygenin. For simultaneous determination of Hb protein and RNA, we used the same FISH procedure as above, sequentially using guinea pig and rabbit antibodies to Hb and fluorescein, respectively, and secondary antibodies to guinea pig and rabbit labeled with Alexa 647 and 488, respectively.

Following fixation and staining, embryos were mounted in 40 ml mounting medium (Prolong Antifade by Invitrogen) and covered with a 22×30 mm cover glass (No. 1½).

### Confocal Microscopy

Following the methods of Janssens et al. [76], whole-embryo images were taken using a laser confocal scanning microscope (Leica TCS SP2). Images were collected using an HC PL APO 20× objective and variable digital zoom (1.2–1.5×). Fluorophores were excited by laser at different wavelengths (488, 555, and 647 nm), and detected via a filterless spectral separation system. Channels were scanned sequentially. To reduce image noise from the photomultiplier tubes, each embryo was scanned sequentially 16 times and the results averaged.

The settings of the microscope were adjusted for each gene product such that pixels expressed at maximum intensity were 255 on the 8-bit scale. Initial image size before processing was 1024×1024 pixels. Raw images were averaged, cropped and rotated. This standardization allowed us to compare levels of gene expression at different times, or in different experiments performed on different days [77].

### Processing of Images

For embryos triply-stained for segmentation proteins, the extraction of AP intensity profiles is well established [76],[77]. With such data, a nuclear mask can be created, and intensity data mapped to nuclei (next section). Co-staining for Bcd and Hb proteins and β-galactosidase mRNA presents greater challenges: signal strength and quality are very different for proteins and RNA; and the anterior localization of Bcd and Hb make identification of posterior nuclei very difficult. We developed a non-mask method for profile extraction for these experiments (section after next).

### Image Processing via Nuclear Masks

For embryos stained for three segmentation proteins, the three images are used to generate a ‘pixel maximum’ image, of the brightest pixels among the images. On this image, pixels are then classified as belonging to a nucleus or not, by edge-detection of bright nuclei against dark background. An error-correction step repairs any ‘fused’ nuclei. With the resulting nuclear mask, dorso-ventral, AP coordinates, and average fluorescence level of the three gene products can be mapped to individual nuclei. Intensity profiles are extracted from a central 10% strip of nuclei along the AP axis [76],[77].

### Direct Image Processing (No Nuclear Mask)

For lacZ embryos, co-stained for Hb and Bcd proteins and β-galactosidase mRNA, nuclei cannot be reliably identified, especially in the posterior (preliminary nuclear staining in a fourth channel shows much crosstalk). For these experiments, we directly extract the pixel intensities in a 10% strip (corresponding area to above). For high-intensity protein staining the signal is strong, but for low-intensity RNA staining we must recover expression from a noisy signal (next section). A one dimensional (1D) AP profile was created from the strips, by averaging intensities in each DV pixel column from the central 10% strip along the AP axis. In addition to some between-pixel noise, the resulting profiles show noise in nuclear order and in the distribution of stained material between nuclei and cytoplasm. Minimization of these two sources of noise is described in the next section. To test the quality of our direct method, we manually made nuclear masks for several co-stained lacZ embryos using the multiple ROI feature in *ImageJ* software [78]. ROIs are circles with radii comparable to the nuclear radii in a given image. Each ROI was positioned manually to outline a given nucleus. Nuclear-resolution AP profiles from this method are of comparable quality to pixel-resolution profiles from our direct extraction method.

### Signal–Noise Decomposition

Noise in intensity profiles can influence model-fitting and statistical analysis of expression patterns [77],[78]. To obtain clear expression patterns, we used singular spectrum analysis (SSA [79]), a non-parametric technique with an adaptive filter. This allowed us to remove experimental (e.g., photomultiplier tube) noise and noise due to variability in nuclear order and in nuclear-cytoplasmic distribution of gene products. We used the methods of Golyandina et al [80], and software developed by Nina Golyandina and Theodore Alexandrov [81].

### Background Removal

Non-specific binding of antibodies to biological material results in background fluorescence in our images. For triple-stained protein images it has been shown [82] that this background is a paraboloid. For every image we calculated the parameters of this paraboloid from regions of the embryo in which a particular gene is not expressed, then transformed original fluorescence at or below this background to zero. For lacZ embryos simultaneously stained for protein and mRNA it is unclear whether background has a comparable shape; in these cases, we use a simple flat background, subtracting the minimum raw intensity off all values.

### Quantifying Border Position and Sharpness

An advantage of direct image processing is the large number of data points (around 1000) and smoothness of each profile. This makes it possible to apply standard calculus techniques to characterize the profiles: we define the Hb domain border as the inflection point, and sharpness as the first derivative at that position. With normalized intensity data (0–100% scale), this slope can be expressed as an angle of inclination (as in Figure 2). These techniques can be applied to data, as well as HSR model results.

### Temporal Classification

In addition to confocal scanning, all embryos were observed along the dorsal edge with Differential Interference Contrast (DIC) optics. Distances were measured from the egg surface to the invaginating membrane, and from the surface to the cortex. The ratio of membrane depth to cortex depth was used to estimate embryo age in minutes, using a published standard curve [63].

### The Zero Eigenvalue Analysis

The Zero Eigenvalue Analysis [56]–[58] is a very efficient method, because the search for bistability is reduced to the solution of a system of equalities and inequalities (see Equation S2.22 in Text S2) that are easier to find than a direct solution of the polynomial equation describing the stationary states (see Equations S1.3′–9′, S1.15′, and S1.16′). This technique readily allows one to find the set of kinetic parameters that produce bistability, and gives two steady state solutions, which can be used to easily make the bifurcation diagram, like that shown in Figure 4. Finding bistability with direct solution of a polynomial requires solutions that are different, real and positive. This is frequently not convenient for degree higher than 2 [83], and not analytically solvable for degree higher than 4. Zero Eigenvalue Analysis can be applied to such higher degree systems. For example, Li [58] has used this method to determine multiplicity of stationary states in the famous Goldbeter and Lefever allosteric model [84], consisting of 14 species, 32 reactions, and 27 kinetic constants.

## Supporting Information

Table S1 Kinetic constants for model fitting and simulations.
(0.02 MB PDF)

Table S2 Kinetic constants to simulate a weak and strong response to half-dosage of *bcd* ^K57R^.
(0.01 MB PDF)

Text S1 Model equations and fitting procedure.
(0.03 MB PDF)

Text S2 Analysis for multiple stable stationary states.
(0.14 MB PDF)

Text S3 Obtaining the logarithmic Hill equation.
(0.02 MB PDF)

Figure S1 Some individual embryo images for the profiles in Figure 1. Each of the overlays in Figure 1D–H is composed of intensity profiles, along the AP axis, from individual embryo images. Here, we show some examples of these individuals, with expression patterns for *hb*; individual embryos on top and profiles on bottom. (A) An embryo homozygous for *hb^14F^*, one of the profiles used in Figure 1D. (B) An embryo from the overlay in Figure 1E. (C) An embryo with a half dosage of Bicoid (Bcd) mRNA (*bcd^E1^*/+; see Figure 1F); (D) An embryo with a weak *bcd^K57R^* phenotype (see Figure 1G); (E) An embryo with a strong *bcd^K57R^* phenotype (see Figure 1H).
(1.59 MB TIF)

Figure S2 Non-normalized profiles for 39 embryos expressing the *hb^14F^* allele. The *hb^14F^* homozygotes can be easily identified by low signal intensities, as described in the literature [53]. WT and heterozygotes could not be easily distinguished in this way.
(0.55 MB TIF)

Figure S3 The influence of experimental error on sharpness measurement. Δ*y*_ex_ is the variability due to staining procedure, such as embryo fixation and permeabilization, and Δ*x* is the projection on the AP axis. Even though P1 and P2 have the same AP projection (Δ*x*) they will have different sharpness measurements (β>α), but this difference is caused by the experimental error Δ*y*_ex_. With normalized patterns (Δ*y*_ex_ = 0 and Δ*y* = 1), sharpness measurement will be determined only by the AP projection.
(0.18 MB TIF)

Figure S4 Hb promoter and lacZ artificial construct. A fragment of 4776 bp from the *hb* gene. Hb (blue) and Bcd (red) sites were identified by DNAse footprinting in [35] and [43], respectively. Black arrows indicate the transcription initiation sites. Green arrows indicate the position of the fragment used in the pThb5 lacZ construct, which includes the six Bcd and both Hb sites. To avoid an unnecessarily extensive model, we took into account only two Hb sites.
(0.26 MB TIF)

Figure S5 Loss of sharpness shown by FISH images for the mRNA expression pattern of the pThb5 lacZ construct, driven by a six Bcd and two Hb site promoter. (A) An individual embryo on top and its corresponding profile on bottom. (B) Overlay of 9 profiles. The pattern shows a slight anterior shift (45.7% EL from 47% EL in WT) and 9.3% decrease in sharpness (72.7° from 80.2° in WT) Heavy line: Model simulation taking into account both Hb sites on this construct (6B2H_lacZ); pattern positioned at 48.0% EL with 74.9° inclination.
(0.50 MB TIF)

Figure S6 Demonstrating reduced sharpness in the pThb5 lacZ construct with the alternate method of enzymatic in-situ hybridization (compare to FISH, used in Fig. S5). (A) Embryo image taken by Differential Interference Contrast (DIC) optics on top and corresponding intensity profile on bottom. (B) 21 profiles taken by the same procedure. Pattern position gives 47.3% EL and 74.9° sharpness. Heavy blue line is the simulation of HSR model (6B2H_lacZ) model for this construct (48.0% EL and 74.9° inclination). The observed reduction in sharpness is independent of the method used for visualizing mRNA.
(0.63 MB TIF)

Figure S7 Temporal evolution of the Hb pattern. Multicolored and dark blue lines indicate experimental data and HSR model, respectively. Embryos are the same as in Figure 3B–D. The embryo ages are indicated in the legend. The oldest embryo, 36.4 min, is the same used to fit the model (Fig. 3A). Each plot covers a temporal window of 9.1 min: (A) Embryos having 9.1 minutes or less in cycle 14; (B), (C) and (D) show embryos having ages from 9.2 to 18.2, 18.3 to 27.3 and 27.4 to 36.4 min, respectively. The earliest computed pattern corresponds to age 2.3 min. There is an interval of roughly 2.3 min between each computed pattern; four of them are shown in each plot. See Materials and Methods for embryo temporal classification.
(1.16 MB TIF)

Figure S8 *hb* RNA and protein profiles. Simultaneous fluorescence immunostaining and in situ hybridization showing *hb* RNA and protein. (A) RNA (above) and protein (below) signals from two different embryos (right and left); (B) RNA (solid lines) and protein (dashed lines) from left (blue) and right (red) embryos in (A), respectively. (C) Comparison between 13 RNA and protein patterns, left and middle chart, respectively. The difference in sharpness is indicated, on right chart, by two straight lines having inclinations of 84.2° (red) and 82.5° (black), which correspond to RNA and protein mean inclinations, respectively (see Table 1). The high sharpness for RNA data supports our result that Hb sharpness results from spatially bistable dynamics.
(1.11 MB TIF)

Figure S9 The HSR model is robust for different Hb initial concentrations. Running the HSR model with an initial condition of zero Hb concentration (yellow line) or a Hb pattern taken from cycle 13 (gray line) gives the same result (solid blue and dotted red lines, respectively), indicating no effect from different initial conditions. This is in agreement with previous results reporting that embryos lacking maternal Hb develop normally [42], [65]–[66].
(0.14 MB TIF)

Figure S10 Bistability diagram showing the origin of Hb robustness for different initial conditions. Green line on the Bcd concentration axis indicates the bistable region, where for each Bcd concentration there are three stationary states available for Hb concentration. Blue and red lines are the stable and unstable branches of stationary states, respectively. Black circles indicate initial conditions outside the bistable region (green line); they evolve to the upper stable branch. Red and green circles indicate initial conditions inside the bistable region: All initial Hb conditions below the unstable branch (red circles) evolve to the lower stable branch, having low Hb concentrations; and initial Hb concentrations above the unstable branch (green circles) evolve to the upper stable branch, having high Hb concentrations. The arrows indicate the temporal evolution.
(0.22 MB TIF)
