# Supplementary material for: Spatial Bistability Generates hunchback Expression Sharpness in the Drosophila Embryo
Source: PLoS Comput Biol. 2008 Sep 26;4(9):e1000184. doi: 10.1371/journal.pcbi.1000184 (PMC2527687; doi:10.1371/journal.pcbi.1000184)
Supplement: Table S1 — Kinetic constants for model fitting and simulations. (0.02 MB PDF) [file pcbi.1000184.s001.pdf]

Table S1. Parameters for model fitting and simulations

The kinetic constants and diffusion coefficients were determined by fitting the *hb* self-regulatory (HSR) model to a pair of Bcd and Hb expression patterns (Fig 3A; see Text S1). These parameters were then used to simulate WT development (Fig 3B). Constant k_1, for Bcd production, was non-zero only at 9 % EL, the position of maximum intensity on the Bcd profile. We used zero initial $[B]_T$. We introduced cooperativity by taking $k_{b(n-1),bn} = factor^n.k_{b0,b1}$ and $k_{bn,b(n-1)} = k_{b1,b0}$ for $n = 2, \ldots, 6$. In order to reduce the number of free parameters and reproduce the effects of different numbers of transcriptional factors binding the promoter region, we make the binding of an additional Bcd molecule to the *hb* promoter increase Hb synthesis (reactions r_{1+2n}, $n = 1, \ldots, 6$) by a constant factor (*Synt_Factor*). We did that by applying the relation $k_{bn,H} = (1 + Synt\_Factor).k_{b(n-1),H}$ for $n = 2, \ldots, 6$. For all simulations, Bcd and Hb diffusion coefficients are 1.9988e-2 and 4.0011e-4, respectively, and total promoter concentration is 5.3430e-1. All parameters are presented in arbitrary units (AU).

Reaction<br>Kinetic Constants		
$0 \rightarrow B$<br>$k_{0,B}$ = 1.9361e+3	$b_3 \rightarrow b_3 + H$<br>$k_{b3,H}$ = 1.1060e+00	$b_6 \rightarrow b_6 + H$<br>$k_{b6,H}$ = 2.2659e+00
$B + b_0 \rightarrow b_1$<br>$k_{b0,b1}$ = 2.4162e-4	$B + b_3 \rightarrow b_4$<br>$k_{b3,b4}$ = 3.9876e-03	$H + h_0 \rightarrow h_1$<br>$k_{h0,b1}$ = 4.4817e-02
$b_1 \rightarrow B + b_0$<br>$k_{b1,b0}$ = 8.7306e-2	$b_4 \rightarrow B + b_3$<br>$k_{b4,b3}$ = 8.7306e-02	$h_1 \rightarrow H + h_0$<br>$k_{h1,h0}$ = 7.8950e-01

$b_1 \rightarrow b_1 + H$ $k_{b1,H} = 3.3272e\text{-}1$	$b_4 \rightarrow b_4 + H$ $k_{b4,H} = 1.4926e{+}00$	$h_1 \rightarrow h_1 + H$ $k_{h1,H} = 7.3846e{+}00$
$B + b_1 \rightarrow b_2$ $k_{b1,b2} = 6.1516e\text{-}4$	$B + b_4 \rightarrow b_5$ $k_{b4,b5} = 1.0152e\text{-}02$	$H + h_1 \rightarrow h_2$ $k_{h1,h2} = 1.7652e{+}01$
$b_2 \rightarrow B + b_1$ $k_{b2,b1} = 8.7306e\text{-}02$	$b_5 \rightarrow B + b_4$ $k_{b5,b4} = 8.7306e\text{-}02$	$h_2 \rightarrow H + h_1$ $k_{h2,h1} = 5.9402e\text{-}01$
$b_2 \rightarrow b_2 + H$ $k_{b2,H} = 7.1935e\text{-}01$	$b_5 \rightarrow b_5 + H$ $k_{b5,H} = 1.8792e{+}00$	$h_2 \rightarrow h_2 + H$ $k_{h2,H} = 1.9021e{+}01$
$B + b_2 \rightarrow b_3$ $k_{b2,b3} = 1.5662e\text{-}03$	$B + b_5 \rightarrow b_6$ $k_{b5,b6} = 2.5848e\text{-}02$	$H \rightarrow 0$ $k_{H,0} = 7.0823e{+}00$
$b_3 \rightarrow B + b_2$ $k_{b3,b2} = 8.7306e\text{-}02$	$b_6 \rightarrow B + b_5$ $k_{b6,b5} = 8.7306e\text{-}02$	$B \rightarrow 0$ $k_{B,0} = 3.1164e\text{-}01$
