# Supplementary material for: Spatial Bistability Generates hunchback Expression Sharpness in the Drosophila Embryo
Source: PLoS Comput Biol. 2008 Sep 26;4(9):e1000184. doi: 10.1371/journal.pcbi.1000184 (PMC2527687; doi:10.1371/journal.pcbi.1000184)
Supplement: Table S2 — Kinetic constants to simulate a weak and strong response to half-dosage of bcd K57R. (0.01 MB PDF) [file pcbi.1000184.s002.pdf]

Table S2. Kinetic constants to simulate a weak and strong response to half-dosage of *bcd*^{K57R}

The simulations for weak and strong response to half-dosage of *bcd*^{K57R} are the heavy blue lines in Fig. 1G and 1H, respectively. All other parameters as in Table S1. This value of $k_{0,B}$ was also used to simulate WT Bcd half-dosage (*bcd*^{E1}/+; heavy blue line in Fig. 1F).

Strong K57R	Weak K57R
$k_{0,B}$ = 1.2908+03	$k_{0,B}$ = 1.2908e+03
$k_{b1,b2}$ = 4.3677e-04	$k_{b1,b2}$ = 5.8440e-04
$k_{b2,b3}$ = 7.8953e-04	$k_{b2,b3}$ = 1.4135e-03
$k_{b3,b4}$ = 1.4272e-03	$k_{b3,b4}$ = 3.4189e-03
$k_{b4,b5}$ = 2.580e-03	$k_{b4,b5}$ = 8.2693e-03
$k_{b5,b6}$ = 4.6637e-03	$k_{b5,b6}$ = 2.0001e-02
