# Supplementary material for: Spatial Bistability Generates hunchback Expression Sharpness in the Drosophila Embryo
Source: PLoS Comput Biol. 2008 Sep 26;4(9):e1000184. doi: 10.1371/journal.pcbi.1000184 (PMC2527687; doi:10.1371/journal.pcbi.1000184)
Supplement: Text S1 — Model equations and fitting procedure. (0.03 MB PDF) [file pcbi.1000184.s003.pdf]

Text S1. Model equations and fitting procedure

In the *hunchback* self-regulatory (HSR) model, the total Bcd and Hb concentrations, free or bound to DNA, are given by:

$$[B]_T = [B] + [b_1] + 2.[b_2] + 3.[b_3] + 4.[b_4] + 5.[b_5] + 6.[b_6] \quad \text{(S1.1)}$$

$$[H]_T = [H] + [h_1] + 2.[h_2] \quad \text{(S1.2)}$$

Brackets denote concentration, in arbitrary units (AU).

Applying the Law of Mass Action [1] to the HSR model (Fig. 2) we obtain a set of ordinary differential equations that describes the temporal evolution of the concentrations due to the reactions, as follows:

$$d[H]/dt = k_{h1,H}.[h_1] + k_{h1,h0}.[h_1] - k_{h0,b1}.[H].[h_0] - k_{h1,h2}.[H].[h_1] + k_{b5,H}.[b_5]$$
$$+ k_{h2,h1}.[h_2] - k_{H,0}.[H] + k_{h2,H}.[h_2] + k_{b1,H}.[b_1] + k_{b2,H}.[b_2] + k_{b3,H}.[b_3]$$
$$+ k_{b4,H}.[b_4] + k_{b6,H}.[b_6] \quad \text{(S1.3)}$$

$$d[h_0]/dt = k_{h1,h0}.[h_1] - k_{h0,b1}.[H].[h_0] \quad \text{(S1.4)}$$

$$d[h_1]/dt = - k_{h1,h0}.[h_1] + k_{h0,b1}.[H].[h_0] - k_{h1,h2}.[H].[h_1] + k_{h2,h1}.[h_2] \quad \text{(S1.5)}$$

$$d[h_2]/dt = k_{h1,h2}.[H].[h_1] - k_{h2,h1}.[h_2] \quad \text{(S1.6)}$$

$$d[B]/dt = k_{b3,b2}.[b_3] + k_{b5,b4}.[b_5] + k_{b2,b1}.[b_2] + k_{b1,b0}.[b_1] - k_{B,0}.[B] - k_{b4,b5}.[B].[b_4]$$
$$- k_{b5,b6}.[B].[b_5] - k_{b0,b1}.[B].[b_0] - k_{b1,b2}.[B].[b_1] - k_{b2,b3}.[B].[b_2]$$
$$- k_{b3,b4}.[B].[b_3] + k_{b4,b3}.[b_4] + k_{b6,b5}.[b_6] + k_{0,B} \quad \text{(S1.7)}$$

$$d[b_0]/dt = k_{b1,b0}.[b_1] - k_{b0,b1}.[B].[b_0] \quad \text{(S1.8)}$$

$$d[b_1]/dt = k_{b2,b1}.[b_2] - k_{b1,b0}.[b_1] + k_{b0,b1}.[B].[b_0] - k_{b1,b2}.[B].[b_1] \quad \text{(S1.9)}$$

$$d[b_2]/dt = k_{b3,b2}.[b_3] – k_{b2,b1}.[b_2] + k_{b1,b2}.[B].[b_1] – k_{b2,b3}.[B].[b_2] \quad (S1.10)$$

$$d[b_3]/dt = – k_{b3,b2}.[b_3] + k_{b2,b3}.[B].[b_2] – k_{b3,b4}.[B].[b_3] + k_{b4,b3}.[b_4] \quad (S1.11)$$

$$d[b_4]/dt = k_{b5,b4}.[b_5] – k_{b4,b5}.[B].[b_4] + k_{b3,b4}.[B].[b_3] – k_{b4,b3}.[b_4] \quad (S1.12)$$

$$d[b_5]/dt = – k_{b5,b4}.[b_5] + k_{b4,b5}.[B].[b_4] – k_{b5,b6}.[B].[b_5] + k_{b6,b5}.[b_6] \quad (S1.13)$$

$$d[b_6]/dt = k_{b5,b6}.[B].[b_5] – k_{b6,b5}.[b_6] \quad (S1.14)$$

With the conservation equations:

$$[b_0] + [b_1] + [b_2] + [b_3] + [b_4] + [b_5] + [b_6] = b_{initial} \quad (S1.15)$$

$$[h_0] + [h_1] + [h_2] = h_{initial} \quad (S1.16)$$

where $b_{initial}$ and $h_{initial}$ are the promoter concentrations, taken as identical constants.

Taking into account the spatial distribution of concentrations along the AP axis and allowing for protein diffusion (Fick’s Law) produces a system of partial differential equations:

$$\frac{\partial X_i(s,t)}{\partial t} = D_{X_i} \frac{\partial^2 X_i(s,t)}{\partial s^2} + F(X_i) \qquad (i = 1, 2, \ldots, 12) \quad (S1.17)$$

where X_i is the concentration of species i (12 species); s and t are the space and time variables, respectively; D_{Xi}, the diffusion coefficient of species X_i, is null for all species except for the free proteins B and H. $F(X_i)$ is determined by the time variation in X_i due to reactions (Eq. S1.3-14).

In order to fit the model to experimental Bcd and Hb data, we used a simple gradient descent method where we minimized an error function (vector distance) defined by

$$error = \left[\sum_{j=1}^{100} \left([B]_j^E - [B]_j^T \right)^2 \right]^{\frac{1}{2}} + \left[\sum_{j=1}^{70} \left([H]_j^E - [H]_j^T \right)^2 \right]^{\frac{1}{2}} \qquad \text{(S1.18)}$$

where j is the number of positions along the AP axis; $[B]_j^E$ and $[H]_j^E$ are the experimental intensity levels for Bcd and Hb, respectively; $[B]_j^T$ and $[H]_J^T$ are the corresponding theoretical Bcd and Hb concentrations. These values are calculated from Eqs. (S1.1-S1.2), respectively, which in turn are calculated by solving the 12 equations represented in Eq. (S1.17) with a finite difference technique. Optimization proceeded by calculating $[B]_j^T$ and $[H]_j^T$ changing all kinetic constants by a constant factor (each kinetic constant defines a direction); solving Eqs. (S1.1-S1.2) again, comparing the relative reduction in the error function (Eq. (S1.18)), and choosing the direction of change to generate the largest error minimization. This process was repeated until error was no longer reduced by parameter change. Then the kinetic constants were changed again, by a smaller factor. This variation factor was repeatedly reduced.

The standard deviation for Bcd fitting can be calculated by dividing the first term on the right in (Eq. S1.18) by the squared root of the number of points in the data set for Bcd; for Hb fitting, the standard deviation can be found by dividing the second term on the right by the squared root of the corresponding number of data points for Hb. The fitting procedure was implemented in Matlab software.

Reference

1. Horn F, Jackson R (1972) General mass action kinetics. Archive for Rational Mechanics and Analysis 47: 81- 116.
