# Supplementary material for: Spatial Bistability Generates hunchback Expression Sharpness in the Drosophila Embryo
Source: PLoS Comput Biol. 2008 Sep 26;4(9):e1000184. doi: 10.1371/journal.pcbi.1000184 (PMC2527687; doi:10.1371/journal.pcbi.1000184)
Supplement: Text S2 — Analysis for multiple stable stationary states. (0.14 MB PDF) [file pcbi.1000184.s004.pdf]

Text S2. Analysis for multiple stable stationary states

We used Zero Eigenvalue Analysis [1] to verify if the HSR model exhibits multiple stationary states (particularly bistability). This method was developed by Hsing-Ya Li [1], based on work developed by Feinberg, Horn and Jackson [2-4]. According to this method, a reaction network can exhibit multiple stable stationary states if and only if certain particular relations are satisfied. These restrictions, called sign compatible relations, are given by a system of algebraic equations and inequalities. The calculation of these relations is described below, preceded by basic definitions [1].

In order to apply the Zero Eigenvalue Analysis to a reaction network, the network must be represented in a vector space. Any reaction (α) in a network involving *n* species can generally be represented in a vector space by

$$\sum_{i=1}^{n} r_{i,\alpha} . X_i \rightarrow \sum_{i=1}^{n} p_{i,\alpha} . X_i \qquad \text{(S2.1)}$$

X_i, the i-th species in the network, can be represented by a canonical column vector where the i-th component is 1 and all others are 0:

$$X_i = \left(\overbrace{0, \ldots, 0}^{(i-1)\text{-elements}}, 1, 0, \ldots, 0 \right)^T \qquad \text{(S2.2)}$$

$r_{i,\alpha}$ and $p_{i,\alpha}$ are the stoichiometric coefficients of species *i*, as reactant or product respectively, in the α reaction. Each reversible reaction is treated as a pair of irreversible ones, where the reactants in one reaction are the products in the other and vice-versa. Using this notation, we can represent a reaction network in an *n*-dimensional vector space as:

$$\mathbf{r}_\alpha . \mathbf{X} \rightarrow \mathbf{p}_\alpha . \mathbf{X} \tag{S2.3}$$

$\mathbf{r}_\alpha$ and $\mathbf{p}_\alpha$ are vectors with components $r_{i,\alpha}$ and $p_{i,\alpha}$, respectively. **X** is the *species vector* with components X_i. The dot "." represents a standard dot product between two vectors. Following this notation, the vector $\mathbf{R}_\alpha = (\mathbf{r}_\alpha - \mathbf{p}_\alpha)$ defines the *reaction vector* of reaction α.

A network *A* is a subnetwork of a network *B* if all reactions in *A* can be found in *B*. For a given network a span subnetwork is defined by a subnetwork containing all irreversible reactions and one (and only one, randomly chosen) reaction of each reversible pair of the original network. The rank (*d*) of a network can be defined as the number of linearly independent reaction vectors we can build from it. This is equal to the rank of the *r* x *n* stoichiometric matrix, *N*, associated to the network and defined by:

$$N = \begin{pmatrix} r_{1,1} - p_{1,1} & r_{1,2} - p_{1,2} & \cdots & r_{1,r} - p_{1,r} \\ r_{2,1} - p_{2,1} & r_{2,2} - p_{2,2} & \cdots & r_{2,r} - p_{2,r} \\ \vdots & \vdots & \ddots & \vdots \\ r_{n,1} - p_{n,1} & r_{n,2} - p_{n,2} & \cdots & r_{n,r} - p_{n,r} \end{pmatrix} \tag{S2.4}$$

where r is the number of reactions in the network. From the matrix above, we can find a subspace called the *stoichiometric subspace* (**S**) defined as a set of linearly independent vectors that we can build from matrix N, using simple procedures from linear algebra.

Two arbitrary vectors **a** and **b**, with components $(a_1, a_2, \ldots, a_n)$ and $(b_1, b_2, \ldots, b_n)$ respectively, are sign compatible if and only if:

$$sign\ a_i = sign\ b_i,\ i = 1, 2, \ldots, n, \text{ where } sign\ a_i \text{ is the sign of } a_i. \tag{S2.5}$$

In order to verify if a network has the ability to exhibit multiple stationary states we must build a span subnetwork, as described above; and verify if the system of equations and inequalities, shown below, are satisfied. If not, then the network cannot exhibit bistability, no matter what values are chosen for the kinetic constants. For a network containing n species, r reactions, p pairs of reversible reactions, and stoichiometric matrix having rank d, the sign compatible relations are as follows:

(i) For every pair of reversible reactions (α, α'), where reaction α belongs to the span subnetwork:

$$\sum_{L=1}^{r-p-d} \left[\xi_L \exp(\mathbf{r}_\alpha \cdot \boldsymbol{\mu}) + a_L\right] d_\alpha^{(L)} \tag{S2.6}$$

$$\sum_{L=1}^{r-p-d} \left[\xi_L \exp(\mathbf{p}_\alpha \cdot \boldsymbol{\mu}) + a_L\right] d_\alpha^{(L)} \tag{S2.7}$$

must be sign compatible with (s.c.w.) the vector $(\mathbf{p}_\alpha - \mathbf{r}_\alpha)\cdot\boldsymbol{\mu}$. μ is an arbitrary vector, see below.

(ii) For every irreversible reaction:

$$\sum_{L=1}^{r\text{-}p\text{-}d} \xi_L d_\alpha^{(L)} > 0 \qquad \text{and} \qquad \sum_{L=1}^{r\text{-}p\text{-}d} \left[\xi_L \exp(\mathbf{p}_\alpha \cdot \boldsymbol{\mu}) + a_L\right] d_\alpha^{(L)} = 0 \tag{S2.8}$$

Parameters a and ξ are arbitrary and real.

Two additional restrictions must be satisfied. The arbitrary vector **μ** must be s.c.w. the stoichiometric subspace **S**, i.e. there must exist a vector **σ**, belonging to **S**, which is s.c.w. vector **μ**. Besides this, parameters $d_\alpha^{(L)}$ must be determined from the following equation:

$$\sum_{\alpha}^{r\text{-}p} d_\alpha^{(L)}(\mathbf{r}_\alpha - \mathbf{p}_\alpha) = \mathbf{0}, \quad L = 1, 2, ..., r - p - d \tag{S2.9}$$

Vectors $\mathbf{r}_\alpha$ and $\mathbf{p}_\alpha$ are defined as above (Eq. S2.3), and must be obtained from a span network that must be chosen from the original network under analysis.

If the relationships above are satisfied, then the solution (**μ**, ξ_L, a_L, L=1, 2, ..., $r - p - d$) can be used to build two stationary states, i.e, two set of values for $\{X_1', X_2', ..., X_n'\}$ and $\{X_1'', X_2'', ..., X_n''\}$ for the concentration of the n species in the network. The concentration of each species can be determined by:

$$X_j' = X_j'' + \sigma_j \ , \qquad j = 1, 2, ..., n \tag{S2.10}$$

$$X_j'' = \begin{cases} \dfrac{\sigma_j}{\left[\exp(\mu_j) - 1\right]} & \text{if} \quad \mu_j \neq 0 \\ \text{any real positive number,} & \text{if} \quad \mu_j = 0 \end{cases} \tag{S2.11}$$

where $\boldsymbol{\sigma}$ is a arbitrary vector that belongs to the stoichiometric subspace ($\boldsymbol{\sigma} \in \mathbf{S}$) and is s.c.w. $\boldsymbol{\mu}$ i.e. *sign* σ_j = *sign* μ_j , $\forall$ j.

In order to determine which kinetic constants, k_α, can generate the above stationary concentrations we can use the following equations:

$$k_\alpha = \frac{\kappa_\alpha}{\prod_{j=1}^{n} \left(X_j''\right)^{r_{\alpha j}}} \ , \qquad \forall \alpha \tag{S2.12}$$

where $r_{\alpha j}$ is the stoichiometric coefficient of the j-th species in the reaction α. Denominators κ_α can be determined from below relations.

For all irreversible reactions:

$$\kappa_\alpha = \sum_{L=1}^{r-p-d} \xi_L d_\alpha^{(L)} \tag{S2.13}$$

For every pair of reversible reactions (α,α’), where α belongs to the span subnetwork and

$\mathbf{r}_\alpha \cdot \boldsymbol{\mu} \neq \mathbf{p}_\alpha \cdot \boldsymbol{\mu}$:

$$\kappa_\alpha = \frac{\sum_{L=1}^{r-p-d} \left[\xi_L \exp(\mathbf{p}_\alpha \cdot \boldsymbol{\mu}) + a_L\right] d_\alpha^{(L)}}{\exp(\mathbf{p}_\alpha \cdot \boldsymbol{\mu}) - \exp(\mathbf{r}_\alpha \cdot \boldsymbol{\mu})} \tag{S2.14}$$

$$\kappa_{\alpha'} = \frac{\sum_{L=1}^{r-p-d} \left[\xi_L \exp(\mathbf{r}_\alpha \cdot \boldsymbol{\mu}) + a_L\right] d_\alpha^{(L)}}{\exp(\mathbf{p}_\alpha \cdot \boldsymbol{\mu}) - \exp(\mathbf{r}_\alpha \cdot \boldsymbol{\mu})} \tag{S2.15}$$

For every pair of reversible reactions (α,α'), where α belongs to the span subnetwork and $\mathbf{r}_\alpha \cdot \boldsymbol{\mu} = \mathbf{p}_\alpha \cdot \boldsymbol{\mu}$:

$$\begin{gathered} \kappa_\alpha > 0, \quad \kappa_{\alpha'} > 0, \\ \kappa_\alpha - \kappa_{\alpha'} = \sum_{L=1}^{r-p-d} \xi_L d_\alpha^{(L)} \end{gathered} \tag{S2.16}$$

To use Zero Eigenvalue Analysis on our model, we simplified the HSR network [5] by reducing the number of Bcd binding sites (removing reactions 4 to 13, Fig. 2). We called this simplified network s_HSR. The bistable behavior we found analytically for the simplified self-regulatory network is also found in numerical solutions of the full model (Fig. 4).

The application of Zero Eigenvalue Analysis must follow three steps:

(i) Build a vector space to represent the network, using Eqs. (S2.1-S2.3);

(ii) Build the span network and use Eqs. (S2.4) and (S2.9) to determine the stoichiometric subspace **S** and parameters $d_{\alpha}^{(L)}$ to obtain the explicit form for the system of equations Eqs. (S2.6)-(S2.8);

(iii) Verify if the above system has solutions and, if so, determine one pair of solutions by using Eq. (S2.10)-(S2.16).

To build a vector space to represent the simplified network, we can define the following species vectors (according to Eq. S2.2):

$$\mathbf{X}_{(h0)} = [1, 0, 0, 0, 0, 0, 0] \qquad \mathbf{X}_{(H1)} = [0, 1, 0, 0, 0, 0, 0]$$
$$\mathbf{X}_{(H11)} = [0, 0, 1, 0, 0, 0, 0] \qquad \mathbf{X}_{(b0)} = [0, 0, 0, 1, 0, 0, 0] \tag{S2.17}$$
$$\mathbf{X}_{(B1)} = [0, 0, 0, 0, 1, 0, 0] \qquad \mathbf{X}_{(B)} = [0, 0, 0, 0, 0, 1, 0]$$
$$\mathbf{X}_{(H)} = [0, 0, 0, 0, 0, 0, 1]$$

The reaction vectors ($\mathbf{R}_{\alpha} = (\mathbf{r}_{\alpha} - \mathbf{p}_{\alpha})$, see Eqs. S2.1-S2.3) can be written as:

$$R_{(H + h0,\, h1)} = [-1, 1, 0, 0, 0, 0, -1] \qquad R_{(h1,\, H + h0)} = [1, -1, 0, 0, 0, 0, 1]$$
$$R_{(H + h1,\, h2)} = [0, -1, 1, 0, 0, 0, -1] \qquad R_{(h2,\, H + h1)} = [0, 1, -1, 0, 0, 0, 1]$$
$$R_{(h1,\, H + h1)} = [0, 0, 0, 0, 0, 0, 1] \qquad R_{(h2,\, h2 + H)} = [0, 0, 0, 0, 0, 0, 1]$$
$$R_{(B + b0,\, b1)} = [0, 0, 0, -1, 1, -1, 0] \qquad R_{(b1,\, B + b0)} = [0, 0, 0, 1, -1, 1, 0] \tag{S2.18}$$
$$R_{(b1,\, b1 + H)} = [0, 0, 0, 0, 0, 0, 1] \qquad R_{(0,\, B)} = [0, 0, 0, 0, 0, 1, 0]$$
$$R_{(B,\, 0)} = [0, 0, 0, 0, 0, -1, 0] \qquad R_{(H,\, 0)} = [0, 0, 0, 0, 0, 0, -1]$$

Using Eq. (S2.4), the stoichiometric matrix N can be easily determined from the vectors above. It can be written as:

$$N := \begin{bmatrix} -1 & 1 & 0 & 0 & 0 & 0 & 0 & 0 & 0 & 0 & 0 & 0 \\ 1 & -1 & -1 & 1 & 0 & 0 & 0 & 0 & 0 & 0 & 0 & 0 \\ 0 & 0 & 1 & -1 & 0 & 0 & 0 & 0 & 0 & 0 & 0 & 0 \\ 0 & 0 & 0 & 0 & 0 & 0 & -1 & 1 & 0 & 0 & 0 & 0 \\ 0 & 0 & 0 & 0 & 0 & 0 & 1 & -1 & 0 & 0 & 0 & 0 \\ 0 & 0 & 0 & 0 & 0 & 0 & -1 & 1 & 0 & 1 & -1 & 0 \\ -1 & 1 & -1 & 1 & 1 & 1 & 0 & 0 & 1 & 0 & 0 & -1 \end{bmatrix} \quad \text{(S2.19)}$$

This matrix has rank 5.

A span subnetwork for the simplified network (s_HSR) can be chosen as shown in Fig. S2.1:

$0 \rightarrow B$	(1)	$B + b_0 \rightarrow b_1$	(2)	$b_1 \rightarrow b_1 + H$	(3)
$H + h_0 \rightarrow h_1$	(4)	$h_1 \rightarrow h_1 + H$	(5)	$H + h_1 \rightarrow h_2$	(6)
$h_2 \rightarrow h_2 + H$	(7)	$H \rightarrow 0$	(8)		

Fig. S2.1. Span subnetwork for the s_HSR network. Reactions (2), (4) and (6) above are irreversible reactions from the pair of reversible reactions (2), (14) and (26) in Fig. (2), respectively.

The parameters $d_\alpha^{(L)}$ can be determined by using the vectors $\mathbf{r}_\alpha$ and $\mathbf{p}_\alpha$ into Eq. (S2.9):

$$
\begin{array}{lll}
d^{(1)}_{(H+h0,\,h1)} = 0 & d^{(1)}_{(H+h1,\,h2)} = 0 & d^{(1)}_{(h1,\,H+h1)} = -1 \\
d^{(1)}_{(B+b0,\,b1)} = 0 & d^{(1)}_{(b1,\,b1+H)} = 1 & d^{(1)}_{(H,\,0)} = 0 \\
d^{(2)}_{(H+h0,\,h1)} = 0 & d^{(2)}_{(H+h1,\,h2)} = 0 & d^{(2)}_{(h1,\,H+h1)} = 1 \\
d^{(2)}_{(h2,\,h2+H)} = 0 & d^{(2)}_{(B+b0,\,b1)} = 0 & d^{(2)}_{(b1,\,b1+H)} = 0 \\
d^{(2)}_{(0,\,B)} = 0 & d^{(2)}_{(H,\,0)} = 1 & d^{(3)}_{(H+h0,\,h1)} = 0 \\
d^{(3)}_{(H+h1,\,h2)} = 0 & d^{(3)}_{(h1,\,H+h1)} = -1 & d^{(3)}_{(h11,\,h2+H)} = 1 \\
d^{(3)}_{(B+b0,\,b1)} = 0 & d^{(3)}_{(b1,\,b1+H)} = 0 & d^{(3)}_{(H,\,0)} = 0 \\
d^{(3)}_{(0,\,B)} = 0 & &
\end{array}
\qquad (S2.20)
$$

Vector $\boldsymbol{\mu}$ can be defined by

$$\boldsymbol{\mu} = [\mu_{h0}, \mu_{h1}, \mu_{h2}, \mu_{b0}, \mu_{b1}, \mu_{B}, \mu_{H}] \qquad (S2.21)$$

Using the parameters defined above, the sign compatibility relations, Eq. (S2.6)-(S2.8), can be written as:

$$-\mu_{h0} + \mu_{h1} - \mu_H = 0 \quad -\mu_{h1} + \mu_{h2} - \mu_H = 0 \quad -\mu_{b0} + \mu_{b1} - \mu_B = 0$$

$$\mu_B = 0 \quad 0 < \varepsilon_1 - \varepsilon_2 - \varepsilon_3 \quad 0 < \varepsilon_3 \quad 0 < \varepsilon_2$$

$$0 < \varepsilon_1 \quad \varepsilon_1 e^{\mu h1} + a_1 - \varepsilon_2 e^{\mu h1} - a_2 - \varepsilon_3 e^{\mu h1} - a_3 = 0 \tag{S2.22}$$

$$\varepsilon_1 e^{\mu H} + a_1 = 0 \quad \varepsilon_3 e^{\mu h2} + {}_3 = 0 \quad \varepsilon_2 e^{\mu b1} + a_2 = 0$$

By substitution we can verify that the following set of parameters satisfy the above relations:

$$\mu_B = 0 \quad \mu_{b1} = 0 \quad \mu_{b0} = 0 \quad \varepsilon_1 = 1.7$$

$$\varepsilon_2 = 0.2 \quad \varepsilon_3 = 1.4 \quad a_1 = -2.8 \quad a_2 = -\varepsilon_2 \tag{S2.23}$$

$$a_3 = -2.491931 \quad \mu_H = .498991 \quad \mu_{h2} = .576586$$

$$\mu_{h1} = .077595 \quad \mu_{h0} = -.421396$$

They give:

$$\mu = [-.4213964408, .0775947256, .5765858920, 0, 0, 0, .4989911664] \tag{S2.24}$$

which is s.c.w. the stoichiometric subspace (**S**) associated with the simplified network.

The solution above shows that the network does have bistability. In order to determine two stationary solutions we can define a vector **σ**, just respecting the restriction that it must belong to the stoichiometric subspace ($\sigma \in S$) and must be s.c.w. vector **μ**. So, we have:

$$\boldsymbol{\sigma} = [-1/100, .005, .005, 0, 0, 0, 3/2] \quad \text{(S2.25)}$$

Using Eqs. (S2.10) and (S2.11), we have two solutions for the stationary states:

$$\mathbf{X}' = [.019081, .066970, .011411, .035491, .061970, 4.636364, 3.818182]$$
$$\mathbf{X}'' = [.029081, .061970, .006411, .035491, .061970, 4.636364, 2.318182] \quad \text{(S2.26)}$$

Where the components can be identified by:

$$\mathbf{X} = [X_{h0}, X_{h1}, X_{h2}, X_{b0}, X_{b1}, X_B, X_H] \quad \text{(S2.27)}$$

The 6th component of **X** in (S2.26) is arbitrary, and the 4th and 5th were chosen by respecting the conservation equations:

$$h_0 + h_1 + h_2 = \textit{Total promoter concentration} \quad \text{(S2.28)}$$

$b_0 + b_1 =$ *Total promoter concentration*

To determine the kinetic constants that produce the solution above, we can use relations (S2.12) to (S2.16). So, we have:

$$
\begin{aligned}
&k_{(H + h0 \rightarrow h1)} = .148336 && k_{(h1 \rightarrow H + h0)} = .161369 \\
&k_{(H + h1 \rightarrow h2)} = .069610 && k_{(h2 \rightarrow H + h1)} = 1.559902 \\
&k_{(h1 \rightarrow H + h1)} = 1.613692 && k_{(h2 \rightarrow h2 + H)} = 218.386308 \\
&k_{(B + b0 \rightarrow b1)} = .060771 && k_{(b1 \rightarrow B + b0)} = .161369 \\
&k_{(b1 \rightarrow b1 + H)} = 3.227384 && k_{(0 \rightarrow B)} = .01 \\
&k_{(B \rightarrow 0)} = .002157 && k_{(H \rightarrow 0)} = .733333
\end{aligned}
\qquad \text{(S2.29)}
$$

Where we have arbitrarily chosen:

$$
\begin{aligned}
&\kappa_{(H + h, h1)} = \kappa_{(h1, H + h0)} = \kappa_{(H + h1, h2)} = \kappa_{(h2, H + h1)} = .01 \\
&\kappa_{(B + b0, b1)} = \kappa_{(b1, B + b0)} = \kappa_{(0, B)} = \kappa_{(B, 0)} = .01
\end{aligned}
\qquad \text{(S2.30)}
$$

The set of algebraic equations describing the stationary state of the simplified network, s_HSR, can be found from Eqs. (S1.3)-(S1.9), (S1.15) and (S1.16), with $b_{2,\ldots,6} = 0$ (see Text S1) and all derivatives taken as null:

$k_{h1,H}.[h_1] + k_{h1,h0}.[h_1] – k_{h0,b1}.[H].[h_0] – k_{h1,h2}.[H].[h_1] + k_{b5,H}.[b_5] + k_{h2,h1}.[h_2]$

$– k_{H,0}.[H] + k_{h2,H}.[h_2] + k_{b1,H}.[b_1] = 0$ (S1.3’)

$k_{h1,h0}.[h_1] – k_{h0,b1}.[H].[h_0] = 0$ (S1.4’)

$– k_{h1,h0}.[h_1] + k_{h0,b1}.[H].[h_0] – k_{h1,h2}.[H].[h_1] + k_{h2,h1}.[h_2] = 0$ (S1.5’)

$k_{h1,h2}.[H].[h_1] – k_{h2,h1}.[h_2] = 0$ (S1.6’)

$k_{b2,b1}.[b_2] + k_{b1,b0}.[b_1] – k_{B,0}.[B] – k_{b0,b1}.[B].[b_0] – k_{b1,b2}.[B].[b_1] + k_{0,B} = 0$ (S1.7’)

$k_{b1,b0}.[b_1] – k_{b0,b1}.[B].[b_0] = 0$ (S1.8’)

$k_{b2,b1}.[b_2] – k_{b1,b0}.[b_1] + k_{b0,b1}.[B].[b_0] – k_{b1,b2}.[B].[b_1] = 0$ (S1.9’)

$[b_0] + [b_1] + [b_2] = b_{initial}$ (S1.15’)

$[h_0] + [h_1] + [h_2] = h_{initial}$ (S1.16’)

Substitution confirms that the set of values for the kinetic constants (S2.29) and (X_{h0}, X_{h1}, X_{h2}, X_{b0}, X_{b1}, X_B, X_H) given by **X**’ and **X**”, Eq. (S2.26), satisfy the steady states in the above algebraic system. This demonstrates that the network does exhibit multiple stationary states (in particular, bistability).

References

1. Li HY (1998) Zero eigenvalue analysis for the determination of multiple steady states in reaction networks. Zeitschrift Fur Naturforschung Section a-a Journal of Physical Sciences 53: 171-177.
2. Horn F, Jackson R (1972) General mass action kinetics. Archive for Rational Mechanics and Analysis 47: 81-116.
3. Horn F (1972) Necessary and sufficient conditions for complex balancing in chemical-kinetics. Archive for Rational Mechanics and Analysis 49: 172-186.
4. Feinberg M, Horn FJM (1974) Dynamics of Open Chemical Systems and Algebraic Structure of Underlying Reaction Network. Chemical Engineering Science 29: 775-787.

5. Lopes FJP (2002) Análise da Formação de Padrões no Desenvolvimento da *Drosophila melanogaster* através de Modelos de Redes Complexas [PhD thesis]. Rio de Janeiro: Universidade Federal do Rio de Janeiro. 193 p.
