# Supplementary material for: Spatial Bistability Generates hunchback Expression Sharpness in the Drosophila Embryo
Source: PLoS Comput Biol. 2008 Sep 26;4(9):e1000184. doi: 10.1371/journal.pcbi.1000184 (PMC2527687; doi:10.1371/journal.pcbi.1000184)
Supplement: Text S3 — Obtaining the logarithmic Hill equation. (0.02 MB PDF) [file pcbi.1000184.s005.pdf]

Text S3. Obtaining the logarithmic Hill equation

If Bcd is the only factor regulating Hb production, we can take the fraction of *hb* promoter occupancy (*Fr*) as proportional to the normalized protein concentration, which is equivalent to the normalized signal intensity in immunostaining assays (H/H_{max}, Eq. S3.1), since immunofluorescent staining signal is proportional to protein concentration plus a nonspecific background, as already shown [1].

$$Fr = \frac{H}{H_{max}} \tag{S3.1}$$

The Hill equation can then be written as

$$Fr = \frac{Bcd^n}{Bcd^n + Bcd_{1/2}^n} \tag{S3.2}$$

where $Bcd_{1/2}$ is the Bcd concentration necessary for half maximal *hb* promoter occupancy.

Through simple algebra, the equation above can be rewritten to

$$Fr.Bcd^n + Fr.Bcd_{1/2}^n = Bcd^n \tag{S3.3}$$

and further rearranged to

$$Fr.\mathrm{Bcd}_{1/2}^{\mathrm{n}} = \mathrm{Bcd}^{\mathrm{n}}(1 - Fr) \qquad \text{(S3.4)}$$

Dividing both sides by $\mathrm{Bcd}_{1/2}^{\mathrm{n}}(1 - Fr)$ and taking logarithms gives

$$\mathrm{n} = \frac{\ln\left(\frac{Fr}{1-Fr}\right)}{\ln\left(\frac{\mathrm{Bcd}}{\mathrm{Bcd}_{1/2}}\right)} \qquad \text{(S3.5)}$$

Equation (S3.5) was used in the Discussion regarding calculations using the Hill equation.

Reference

1. Gregor T, Wieschaus EF, McGregor AP, Bialek W, Tank DW (2007) Stability and nuclear dynamics of the bicoid morphogen gradient. Cell 130: 141-152.
