# Supplementary figures and images for: Spatial Bistability Generates hunchback Expression Sharpness in the Drosophila Embryo
Source: PLoS Comput Biol. 2008 Sep 26;4(9):e1000184. doi: 10.1371/journal.pcbi.1000184 (PMC2527687; doi:10.1371/journal.pcbi.1000184)

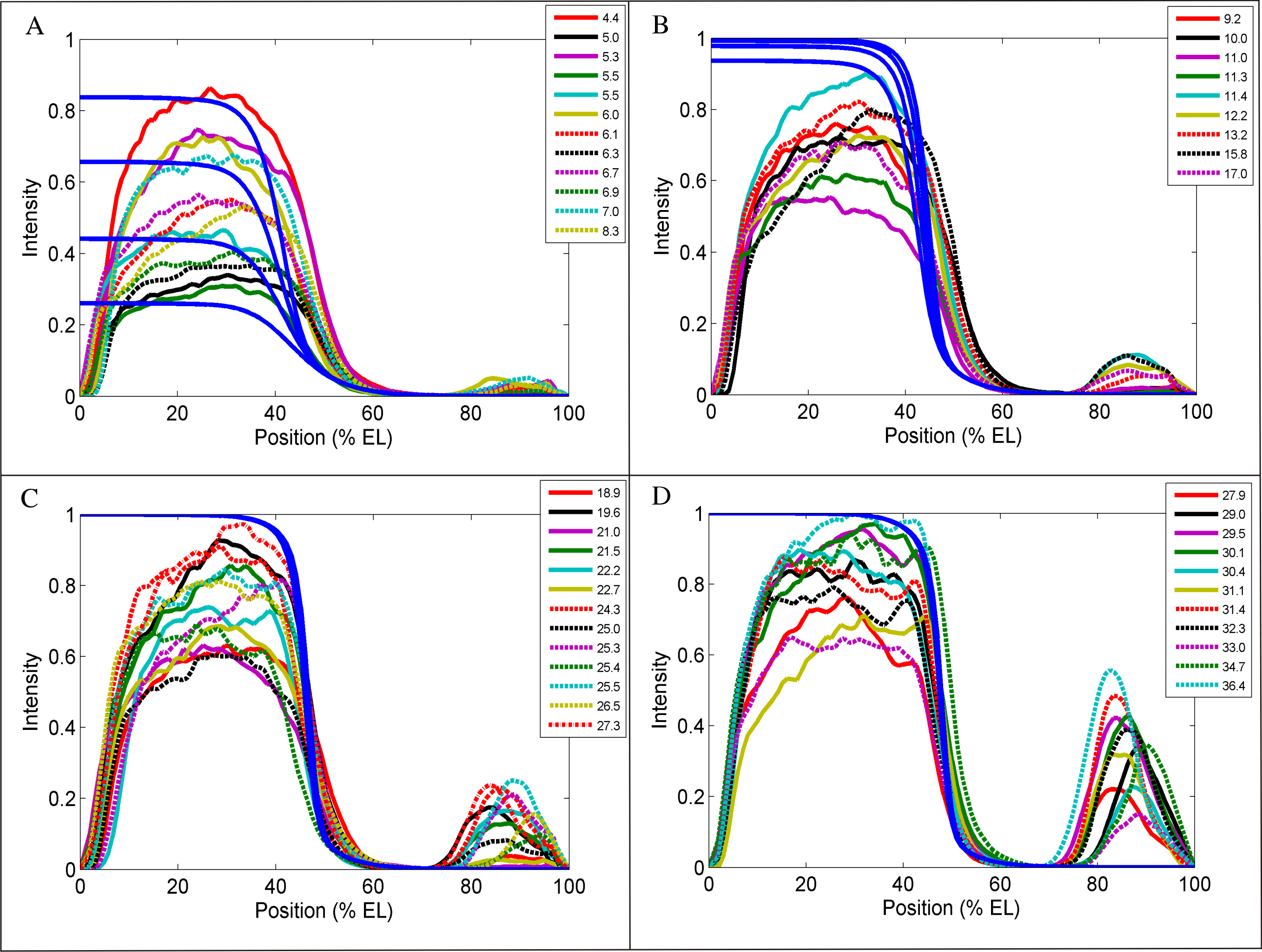

Supplement: Figure S7 — Temporal evolution of the Hb pattern. Multicolored and dark blue lines indicate experimental data and HSR model, respectively. Embryos are the same as in Figure 3B–D. The embryo ages are indicated in the legend. The oldest embryo, 36.4 min, is the same used to fit the model (Fig. 3A). Each plot covers a temporal window of 9.1 min: (A) Embryos having 9.1 minutes or less in cycle 14; (B), (C) and (D) show embryos having ages from 9.2 to 18.2, 18.3 to 27.3 and 27.4 to 36.4 min, respectively. The earliest computed pattern corresponds to age 2.3 min. There is an interval of roughly 2.3 min between each computed pattern; four of them are shown in each plot. See Materials and Methods for embryo temporal classification. (1.16 MB TIF) [file pcbi.1000184.s012.tif]
